# Maximizing Diagnostic Yield in Intellectual Disability Through Exome Sequencing: Genotype–Phenotype Insights in a Vietnamese Cohort

**DOI:** 10.3390/diagnostics15222821

**Published:** 2025-11-07

**Authors:** Thu Lan Hoang, Thi Kim Phuong Doan, Thi Ngoc Lan Hoang, Cam Tu Ho, Thi Ha Vu, Thi Trang Nguyen, Thi Huyen Vu, Thi Trang Dao, Thi Minh Ngoc Nguyen, Phuong Mai Nguyen, Huu Duc Anh Nguyen, Chi Dung Vu, Phuong Thao Do, Quang Phuc Pham, Quang Trung Nguyen, Thi Phuong Mai Nguyen, Thi Thuy Ninh To, Hoa Giang, Thi Lan Anh Luong

**Affiliations:** 1 Department of Medical Biology and Genetics, Hanoi Medical University, Hanoi 100000, Vietnam; hoangthulan@hmu.edu.vn (T.L.H.); doankimphuong@hmu.edu.vn (T.K.P.D.); hoangthingoclan@hmu.edu.vn (T.N.L.H.); vuthiha@hmu.edu.vn (T.H.V.); trangnguyen@hmu.edu.vn (T.T.N.); vuthihuyen@hmu.edu.vn (T.H.V.); trangdao@hmu.edu.vn (T.T.D.); ngocntm0983@hmuh.vn (T.M.N.N.); nguyenphuongmai@hmu.edu.vn (P.M.N.); nguyenhuuducanh@hmu.edu.vn (H.D.A.N.); dungvu@nch.gov.vn (C.D.V.); ninhtohmu@gmail.com (T.T.N.T.); 2 Center of Clinical Genetics and Genomics, Hanoi Medical University Hospital, Hanoi 116177, Vietnam; 3 Center for Gene and Protein Research, Hanoi Medical University, Hanoi 100000, Vietnam; hocamtu@hmu.edu.vn; 4 Center for Endocrinology, Metabolism, Genetics and Molecular Therapy, National Children’s Hospital, Hanoi 100000, Vietnam; 5 Department of Pediatrics, Hanoi Medical University, Hanoi 100000, Vietnam; dophuongthao@hmu.edu.vn (P.T.D.); nguyenthiphuongmai@hmu.edu.vn (T.P.M.N.); 6 Department of Neurosurgery, Thanh Nhan Hospital, Hanoi 100000, Vietnam; bsphamquangphuc@gmail.com; 7 Department of Otolaryngology, Hanoi Medical University, Hanoi 100000, Vietnam; nguyenquangtrung@hmu.edu.vn; 8 Institute of Medical Genetics, Ho Chi Minh 70000, Vietnam; gianghoa@gmail.com

**Keywords:** intellectual disability (ID), whole-exome sequencing (WES), clinical exome sequencing (CES), copy number variants (CNVs), hierarchical cluster analysis (HCA), Z-score, multidimensional phenotypic analysis, genotype–phenotype correlation, pathway clustering, neurodevelopmental disorders

## Abstract

**Background**: Intellectual disability (ID) is a heterogeneous condition caused by diverse genetic factors, including single-nucleotide variants (SNVs) and copy number variants (CNVs). Whole-exome sequencing (WES) and clinical exome sequencing (CES) have become essential tools for identifying pathogenic variants; however, their relative diagnostic performance in ID has not been fully characterized. **Methods**: Children diagnosed with ID or related neurodevelopmental disorders underwent WES or CES. Identified variants were classified according to ACMG/AMP and ClinGen guidelines, with segregation analysis performed when parental samples were available. Diagnostic yields were compared across demographic, prenatal, and phenotypic subgroups. A multidimensional semi-quantitative scoring system encompassing 15 clinical domains (e.g., age at onset, neuro-motor function, seizures, MRI findings, vision, and dysmorphic features) was developed. Z-scores were calculated for each parameter, followed by hierarchical cluster analysis (HCA) and correlation modeling to define genotype–phenotype associations and pathway-level clustering. **Results**: A broad spectrum of pathogenic and likely pathogenic variants across multiple genes and biological pathways was identified in our study. CNV-associated cases frequently exhibited prenatal anomalies or multisystem phenotypes associated with large chromosomal rearrangements. Monogenic variants and their corresponding phenotypic profiles were identified through clinical exome sequencing (CES) and whole-exome sequencing (WES). Phenotypic HCA based on Z-scores revealed three major biological groups of patients with coherent genotype–phenotype relationships: Group 1, severe multisystem neurodevelopmental disorders dominated by transcriptional and RNA-processing genes (*POLR1C, TCF4, HNRNPU, NIPBL, ACTG1*); Group 2, intermediate epileptic and metabolic forms associated with ion-channel and excitability-related genes (*SCN2A, PAH, IQSEC2, GNPAT*); and Group 3, milder or focal neurodevelopmental phenotypes involving myelination and signaling-related genes (*NKX6-2, PLP1, PGAP3, SMAD6, ATP1A3*). Gene distribution significantly differed among these biological categories (χ^2^ = 54.566, df = 34, *p* = 0.0141), confirming non-random, biologically consistent grouping. Higher Z-scores correlated with earlier onset and greater neurological severity, underscoring the clinical relevance of the multidimensional analytical framework. **Conclusions**: This study highlights the genetic complexity and clinical heterogeneity of intellectual disability and demonstrates the superior diagnostic resolution of WES and CES. Integrating multidimensional phenotypic profiling with genomic analysis enhances genotype–phenotype integration and enables data-driven phenotype stratification and pathway-based re-analysis. This combined diagnostic and analytical framework offers a more comprehensive approach to diagnosing monogenic ID and provides a foundation for future predictive and functional studies.

## 1. Introduction

The Diagnostic and Statistical Manual of Mental Disorders, version 5, defines intellectual disability (ID) as neurodevelopmental disorders that begin in childhood and are characterized by significant limitation in conceptual, social, practical skills [1]. Similarly, the International Classification of Diseases 11th revision (ICD-11), also describes disorders of intellectual development (DID) as a condition arising during the developmental period, marked by substantially below-average intellectual functioning and adaptive behavior. These deficits are typically two or more standard deviations below the mean on standardized, individually administered tests, or determined by clinical assessment when formal testing is not feasible. Diagnosis requires evidence of deficits in intellectual functioning (reasoning, problem-solving, planning, abstract thinking, and learning), adaptive functioning in conceptual, social, and practical domains, and onset during the developmental period [2]. ICD 11 characterizes autism spectrum disorder (ASD) as a persistent deficit in reciprocal social communication and interaction, accompanied by restricted, repetitive patterns of behavior, interests, or activities that are atypical or excessive relative to the individual’s age and sociocultural context. Symptom onset occurs during the developmental period, typically in early childhood, although difficulties may not become fully apparent until later. These symptoms result in clinically significant impairment in personal, familial, social, educational, or occupational functioning.

ID affects approximately 1% of the global population and imposes a substantial impact on society [2]. Its etiology is highly heterogeneous, encompassing both genetic and non-genetic factors. Genetic causes account for 30–50% of cases [3] while non-genetic contributors of ID can be grouped into prenatal, perinatal, and postnatal factors [4]. Prenatal causes include maternal infections (e.g., cytomegalovirus, rubella, and Zika virus), teratogenic exposure (e.g., alcohol, antiepileptic drugs, and heavy metals) and maternal comorbidities such as phenylketonuria, pregnancy hypertension, asthma, urinary tract infection, pre-pregnancy obesity, uncontrolled diabetes, malnutrition, and obstetrical complications causing anoxia (placenta previa, placenta abruption, and umbilical cord prolapse) [5,6,7]. Perinatal complication such as hypoxic–ischemic brain injury, extreme prematurity, or low birth weight are also major contributors [8], while postnatal causes include infection (encephalitis, meningitis), head trauma, asphyxia, intracranial tumor, malnutrition and toxin exposure [9]. Although advances in genomic technologies have improved the detection of monogenic and chromosomal etiologies, prevention of non-genetic causes through maternal health organization, infection control, and perinatal care [10].

The genetic landscape of ID is remarkably complex, involving diverse molecular mechanisms. Current diagnostic gene panels include more than 1500 genes and up to 10% of the human genome are thought to influence ID pathogenesis [11,12]. Genotype–phenotype correlations have been established within specific syndromes and across variant types within the same gene. Recent analyses highlight broader molecular clusters with shared cognitive and behavioral features, underscoring ID as a highly heterogeneous group of disorders. Identification of genetic causes enhances understanding of ID pathophysiology while informing clinical prognosis, recurrence risk, and access to condition-specific support programs [13].

Despite many ongoing studies, effective therapies for cognitive impairment remain limited except for a few metabolic disorders. Therefore, understanding the underlying genetic, molecular and cellular mechanisms is essential to identify novel intervention approaches.

In recent years, the advances of chromosomal microarray analysis (CMA) and next-generation sequencing (NGS) technologies have revolutionized ID diagnosis at the molecular level. CMA, recommended as a first-tier clinical test, can detect chromosomal copy number variants (CNVs) and achieve diagnostic yields at approximately 20% for neurodevelopmental disorder etiology [14,15]. For the remaining cases, NGS approach, including whole-exome sequencing (WES) and clinical exome sequencing (CES), is used and collectively explains approximately 35–50% of neurodevelopmental disorder cases [3,16,17].

Beyond sequencing, multidimensional analysis has recently emerged as a complementary strategy to interpret the complex clinical spectrum of intellectual disability. This approach applies semi-quantitative scoring across multiple clinical domains (e.g., onset, motor, neurological, seizure, MRI, vision, dysmorphism, and systemic involvement), followed by Z-score normalization [18] and hierarchical clustering analysis (HCA). By converting qualitative clinical observations into standardized quantitative matrices, multidimensional analysis enables systematic mapping of genotype–phenotype correlation and identification of phenotypic clusters reflecting shared molecular pathway. Such data-driven integration improves the power of genomic findings, facilitates patient stratification for functional studies and supports potential precision medicine applications [19,20,21,22,23,24,25,26].

Leveraging the strengths of NGS and phenotypic clustering, we performed CES and WES on 90 patients with ID and their relatives to elucidate the genetic etiology of ID. This study aimed to evaluate the diagnostic utility of NGS and to explore genotype–phenotype correlations through multidimensional analyses, thereby enhancing our understanding of the molecular and clinical complexity of ID.

## 2. Materials and Methods

**Figure 1:**
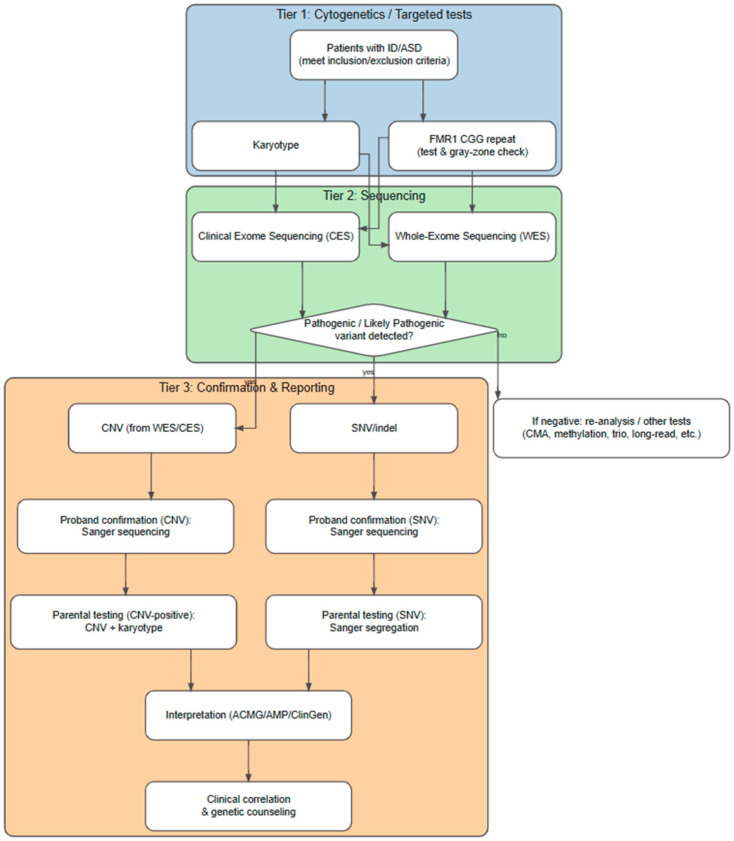
Workflow of genetic testing for patients with intellectual disability (ID) and/or autism spectrum disorder (ASD): Patients meeting the inclusion and exclusion criteria for ID/ASD underwent a stepwise diagnostic workflow. **Tier** **1 (blue):** Cytogenetic and targeted testing, including karyotype analysis and FMR1 CGG repeat expansion testing to detect Fragile X syndrome and gray-zone alleles. **Tier 2 (green):** Exome-based sequencing, using either whole-exome sequencing (WES) or clinical exome sequencing (CES), with detected variants assessed for pathogenicity. **Tier 3 (orange):** Confirmation and reporting, in which pathogenic or likely pathogenic variants were validated using orthogonal methods—Sanger sequencing for SNVs/indels and CNVs—followed by interpretation according to ACMG/AMP/ClinGen guidelines and clinical correlation with genetic counseling. For patients with negative findings, re-analysis or additional testing (e.g., chromosomal microarray, methylation analysis, trio sequencing, or long-read sequencing) was recommended.

This study adopted a stepwise diagnostic strategy to maximize the detection of underlying genetic etiologies in patients presenting with ID and/or ASD. The workflow integrated cytogenetic, sequencing, and confirmatory approaches to ensure comprehensive genomic characterization and accurate clinical interpretation (Figure 1).

All enrolled patients who met the inclusion and exclusion criteria underwent a three-tier genetic testing protocol.

### 2.1. Study Population

The inclusion criteria for patient enrolment were as follows: (1) diagnosis of ID/ASD by a pediatric neurologist or psychiatrist (according to ICD-11 criteria); (2) clinical evaluation and genetic counseling performed by a clinical geneticist and (3) Absence of chromsomal abnormalities associated with ID/ASD and *FMR1* full mutation; (4) received either Exome sequencing (ES) or CES, with ES performed in CES-negative cases to enable broader variant detection.

The exclusion criteria were as follows: (1) patients diagnosed with metabolic diseases based on clinical manifestations and laboratory evaluations; (2) patients whose ID/ASD resulted from perinatal ischemia, hypoxic brain injury, bilirubin encephalopathy, central nervous system infections, poisoning, extreme preterm birth (before 28 weeks of gestation), or trauma; and (3) patients or legal guardians who did not provide consent to participate in the study.

Between January 2023 and January 2025, seventy-five patients from 69 families, diagnosed with ID/ASD of indeterminate etiology, were referred to the Clinical Genetic Center at Hanoi Medical University Hospital by their families for genetic consultation prior to conceiving their next child.

This research is part of the state-level project *“Screening and Etiology Diagnosis of Genetic Mental Retardation in Vietnamese People”* (grant number ĐTĐL.CN-80/22, approved under Decision 2349/QĐ-BKHCN, Hanoi, 2 February 2022) and was conducted with ethical approval (Ethical Certification No. 93/GCN-HĐĐĐNCYYSSH-ĐHYHN, Hanoi, 9 June 2023).

### 2.2. Sample Collection

After obtaining written informed consent from the guardians, 2 mL of peripheral venous blood was collected from each participating child for WES, CES, and CNV detection. In families with multiple affected children exhibiting similar phenotypes, WES/CES was initially performed on a single child. Blood samples were also collected from parents and other siblings for further investigation when a related variant—pathogenic, likely pathogenic, or of uncertain clinical significance (VUS)—was identified in the affected child. Sanger sequencing was employed to confirm monogenic variants and determine their origin (de novo in dominant disorders) or allelic phase (in cis or trans in recessive disorders). Similarly, CNV analysis and karyotyping were performed in parents and relatives to establish the origin of CNV variants.

### 2.3. Whole Exome Sequencing and Clinical Exome Sequencing

Genomic DNA was enzymatically fragmented, and target regions were enriched using DNA capture probes. DNA libraries were prepared with a New England Biolabs kit (Ipswich, MA, USA) and enriched using targeted xGen probes from Integrated DNA Technologies (IDT, Coralville, IA, USA). The enriched libraries were sequenced either for the whole exome (WES) or for 4503 targeted genes (CES) using the Illumina NextSeq System (San Diego, CA, USA), achieving a minimum mean coverage of 100× and at least 95% of target regions covered at >10×. Sequenced reads were aligned to the human reference genome GRCh38 to identify variants.

### 2.4. Copy Number Variant Sequencing (CNVseq)

CNVseq is a NGS–based approach (using WES and CES) designed to identify chromosomal aneuploidies and DNA abnormalities ≥400,000 nucleotides. Quality control of sequencing data was performed with FastQC (version 0.11.9). DNA samples were sequenced on a NextSeq 550 platform using a paired-end 2 × 75 bp Reagent Kit (Illumina, San Diego, CA, USA). Raw sequences were aligned to the human reference genome (GRCh38) using BWA (v0.7.17) and SAMtool(v1.17), and duplicate reads were removed with Picard (v2.27.5) (Broad Institute, Cambridge, MA, USA). The processed data were annotated using the OMIM and DECIPHER databases for CNV analysis.

Variants with a frequency <1% in the Vietnamese genetic database (including the 1000 Exome Sequencing Project) were further analyzed. Variants with a minor allele frequency <1% in gnomAD, as well as deleterious variants reported in the Human Gene Mutation Database (HGMD), ClinVar, or the internal biodata bank, were assessed. Significant variants were localized within coding exons and ±10 nucleotides of splice junctions, supported by definitive genotyping evidence per OMIM^®^ data.

All variants were classified according to ACMG/AMP criteria and ClinGen recommendations into five categories: pathogenic, likely pathogenic, VUS, likely benign, and benign. Variants relevant to the patient phenotype were documented. Variants with low sequence quality or ambiguous genotypes were confirmed by Sanger sequencing, resulting in a specificity exceeding 99.9% for all documented variants. Certain genetic changes—including gene rearrangements (fusions, inversions), short tandem repeat variations, pseudogenes, mosaic variants, and mitochondrial gene variants—may not be detected by this method.

### 2.5. Sanger Sequencing

Gene sequences and mutation locations of interest were obtained from the NCBI Genome database (GRCh38). Primers specific to the target genes were designed using Primer3Plus, evaluated for specificity using NCBI Primer-BLAST (NCBI, Bethesda, MD, USA) and the UCSC In-Silico PCR tool (Santa Cruz, CA, USA), synthesized by IDT, and employed for PCR amplification. PCR was performed on the Applied Biosystems ProFlex™ (Thermo Fisher Scientific, Waltham, MA, USA) 3 × 32-well PCR System using the relevant DNA sequences of the variants as templates. Purified PCR products were subjected to Sanger sequencing on an Applied Biosystems 3130xl Genetic Analyzer with POP-7™ polymer. Sequencing results were analyzed using CodonCode Aligner software (v9.0.1), with alignment and variant verification performed against the UCSC Genome Browser (GRCh38) and NCBI BLAST to determine mutation site and type.

### 2.6. Clinical Phenotyping and Semi-Quantitative Scoring System

Quantitative assessment of clinical severity in genetic and neurodevelopmental disorders has increasingly adopted structured, multi-domain scoring systems to capture phenotypic variability in a standardized and reproducible manner. Previous studies—such as those by Klouwer et al. (2018) in Zellweger spectrum disorders, Aglan et al. (2012) in osteogenesis imperfecta, and the Cornelia de Lange syndrome clinical severity scale—have demonstrated that ordinal scoring across multiple clinical domains provides an objective framework for comparing disease burden and functional outcomes [27,28].

Similarly, Nagy et al. (2022) [29] applied a four-tiered severity scale for POGZ-related neurodevelopmental disorders to classify patients according to overall phenotypic impact. These approaches share key methodological features, including multi-systemic coverage, ordinal grading reflecting progression from mild to severe manifestations, and incorporation of both neurological and systemic parameters—all of which informed the design of the present scoring model [29].

Accordingly, our study employed a 15-domain ordinal scoring system, with each domain rated from 1 (mild or late-onset) to 4 (severe, early-onset, or multisystemic), and an additional “NA/Normal” category for absent or unassessed features. This framework enables consistent quantification of clinical heterogeneity and facilitates genotype–phenotype correlation analyses across the studied cohort. All scores were derived from medical records, clinical examination reports, EEG/MRI findings, and genetic test results, and independently reviewed by two clinical geneticists to minimize subjectivity. This structured scoring system enabled transformation of complex clinical data into quantitative matrices, which were subsequently used for correlation analyses, Z-score normalization, and hierarchical cluster analysis (HCA) to identify phenotypic subgroups and gene–pathway associations (Table 1).

### 2.7. Statistical and Multidimensional Analysis

Descriptive statistics were used to summarize the demographic and clinical characteristics of the cohort. Continuous variables (e.g., age) were reported as mean ± standard deviation (SD), while categorical variables were presented as frequencies and percentages. Comparisons between genetic analysis–positive and –negative patients were performed using independent t-tests for continuous variables and chi-square tests for categorical variables. Statistical significance was defined as *p* < 0.05. All analyses were conducted using SPSS (version 26.0; IBM Corp., Armonk, NY, USA) and R (version 4.4.1; R Foundation for Statistical Computing, Vienna, Austria).

Beyond conventional univariate statistics, an integrated multidimensional analytical framework was developed, building on methodologies previously established in Vietnamese biomedical data analytics [19,20,21,22,23,24,25,26]. These prior studies validated the application of hierarchical clustering, Z-score normalization, and correlation-based multidimensional modeling to investigate complex clinical–genetic interactions.

All quantitative clinical features were standardized using Z-score normalization, defined as

$$Z_{ij}=\frac{X_{ij}-\mu_{j}}{\sigma_{j}}$$

where $X_{ij}$ is the raw value for variable *j* in individual *I*, $\mu_{j}$ is the mean, and $\sigma_{j}$ is the standard deviation for feature *j*. This transformation ensured comparability across heterogeneous clinical scales by rescaling all domains to a mean of 0 and SD of 1. The normalized matrix was subjected to hierarchical cluster analysis (HCA) using Euclidean distance and complete linkage to identify latent groupings of patients sharing similar clinical patterns. Heatmap visualization integrated HCA-derived structure with gene-level and biological pathway annotations (e.g., ion channel regulation, transcriptional control, myelination, and metabolic signaling) to highlight phenotypic convergence and mechanistic differentiation across gene groups.

Pairwise relationships among clinical domains were quantified using Pearson’s correlation coefficients (R) with corresponding *p*-values, visualized in a color-coded correlation heatmap. This matrix-based representation provided insight into interdependent phenotypic features and potential syndromic clusters within the cohort.

## 3. Results

### 3.1. Sex and Age Distribution of Gene Variants

Among 75 patients with ID, 47 were male and 28 were female with ages ranging from 2 to 23 years (mean ± SD: 9.7 ± X years). Of these, 59 underwent WES, 10 underwent CES, and the remaining six affected siblings had their genetic diagnoses confirmed by Sanger sequencing (Supplementary Tables S1–S3).

Causal variants were identified in 38 of 75 patients, comprising 28 cases with pathogenic or likely pathogenic variants linked to monogenic diseases and 10 cases with pathogenic CNVs. Additionally, seven unconfirmed cases carried either variants of unknown clinical significance (VUSs) or a pathogenic heterozygous variant inherited from an unaffected parent.

A small proportion of cases were diagnosed between ages 3 and 6 years (8.3% for CNVs and 5% for SNVs), whereas most CNV cases (41.7%) were identified in children aged 6–8 years. In contrast, SNV-positive cases were predominantly observed in older children, with 67.5% detected in those over 8 years of age. The mean age for CNV cases was 8.5 ± 3.5 years, compared with 10.4 ± 4.4 years for SNV cases, suggesting that CNVs tend to be diagnosed earlier than SNVs, likely due to the more pronounced clinical features associated with larger genomic alterations [11].

Among patients analyzed by CES or WES, the overall molecular diagnostic yield (detection of pathogenic or likely pathogenic variants) did not differ significantly between males and females (Supplementary Figure S1). Although males exhibited a slightly higher positive rate, this difference was not statistically significant (*p* > 0.05, Chi-square or Fisher’s exact test). Similarly, age distribution did not differ between patients with positive and negative genetic findings (Supplementary Figure S2). Median ages were comparable, as confirmed by the Mann–Whitney U test (*p* = 0.598). Age density distribution (Supplementary Figure S3) showed substantial overlap between the two groups, indicating that diagnostic yield was independent of age and that no apparent age-related bias influenced the detection of pathogenic variants.

### 3.2. Monogenic Variants in Children with ID

Among the 28 cases with identified monogenic variants, 11 de novo variants were detected in nine families, along with 15 novel variants. Parental and sibling samples were subsequently analyzed by Sanger sequencing to confirm segregation patterns. All variants were classified according to the ACMG/AMP/ClinGen Sequence Variant Interpretation (SVI) guidelines.

Our analysis highlighted a strong association between neurological impairment, motor development delays, intellectual disability, and dysmorphic features in genetic disorders. Most genes were linked to multiple phenotypic domains, underscoring the importance of comprehensive diagnostic assessment. Syndromic forms of ID, such as Cornelia de Lange (*NIPBL*), Pitt-Hopkins (*TCF4*), and Baraitser-Winter (*ACTG1*), frequently exhibited distinctive craniofacial features along with severe cognitive and motor deficits. In contrast, isolated neurological conditions, such as phenylketonuria (PKU) caused by *PAH* gene mutations, illustrate that some disorders can be effectively managed through early detection and timely intervention.

### 3.3. Visualization and Key Insights

#### 3.3.1. Gene Mutation Frequency and Mutation Type Distribution

*NKX6-2* exhibited the highest mutation frequency, with five confirmed cases from two families, highlighting its potential clinical relevance. *SCN2A* followed, with three confirmed cases from two families, consistent with its well-established role in epilepsy and neurodevelopmental delay. Mutations in *PRUNE1*, *SMAD6*, and *NIPBL* were deteced at lower frequencies, each in a single case. Additionally, variants in *HEPHL1*, *MT-SS2*, and *OSCEP* were identified only in unconfirmed cases, indicating a need for further validation (Supplementary Table S1).

Among the 18 genes identified in confirmed cases, nine were linked to autosomal dominant diseases or syndromes, with nearly all pathogenic variants occurring de novo, except for two instances in which parental samples were unavailable. Of the remaining cases, seven genes were associated with autosomal recessive inheritance, and two with X-linked recessive disorders.

**Figure 2:**
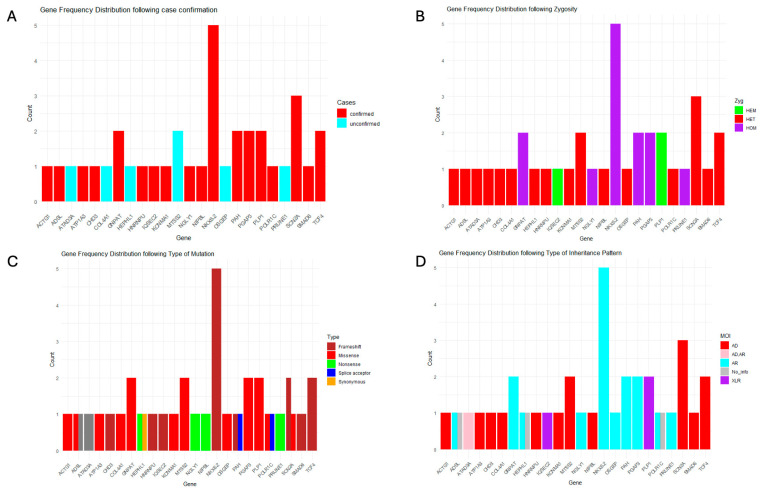
The frequency distribution of gene mutations categorized by case confirmation, zygosity, mutation type, and inheritance pattern. (**A**) Mutation frequency by case confirmation: **red** represents confirmed cases, while **light blue** represents unconfirmed cases. (**B**) Mutation distribution by zygosity: **red** for heterozygous (HET), **green** for hemizygous (HEM), and **purple** for homozygous (HOM). (**C**) Mutation classification by type: **brown** for frameshift, **red** for missense, **green** for nonsense, **blue** for splice acceptor, and **orange** for synonymous mutations. (**D**) Mutation distribution by inheritance pattern: **red** for autosomal dominant (AD), **light blue** for autosomal recessive (AR), **purple** for X-linked recessive (XLR), **rose** for mixed inheritance patterns (AD, AR), and **grey** for no information.

Loss-of-function variants were the most common mutation type, with frameshift variants—arising from insertions and deletions—being the predominant subtype. Missense variants were also prevalent across several genes. Overall, three affected genes were associated with autosomal recessive diseases, whereas four corresponded to autosomal dominant conditions (Figure 2).

#### 3.3.2. Phenotypic Spectra

ID was the predominant clinical feature among the studied patients. Moderate-to-severe ID was observed in individuals carrying variants in *TCF4*, *IQSEC2*, *ADSL*, *NGLY1*, and *PGAP3*. Severe cognitive impairment occurred in patients with mutations in *NKX6-2, PLP1, PAH* gene (when untreated), and *SCN2A* (in early-onset type), whereas milder ID was associated with *SCN2A* (in late-onset type) or *SMAD6* variants.

Distinct dysmorphic features were noted in several cases. For example, a patient with a *NIPBL* mutation (Cornelia de Lange syndrome) presented with synophrys, long curly eyelashes, a depressed nasal bridge, and a thin upper lip, along with vesicoureteral reflux. A patient with an *ATP1A3* mutation (Snijders Blok-Campeau syndrome) exhibited frontal bossing and a round face. A *SMAD6* variant was associated with premature craniosynostosis and hip dislocation, while *TCF4* mutations (Pitt-Hopkins syndrome) produced characteristic facial anomalies, including coarse facial features, a protruding lower face, full cheeks, and cupid’s bow upper lip, in two distinct patients. In contrast, patients with *IQSEC2, KCNN4, ADSL*, or *SCN2A* variants did not display external dysmorphic traits.

Overall, variants in *SCN2A, IQSEC2*, and *TCF4* had pronounced impact on neurological, motor, and cognitive functions across patients. Syndromic conditions, such as Cornelia de Lange, Pitt–Hopkins, and Snijders Blok–Campeau syndromes, were characterized by distinctive facial and skeletal anomalies, whereas other disorders primarily manifested with severe neurological symptoms in the absence of dysmorphic features. Importantly, *PAH* deficiency caused phenylketonuria (PKU), a treatable metabolic disorder, whereas management of other genetic conditions focused on symptomatic and supportive care rather than curative interventions (Table 2, Supplementary Table S2).

**Figure 3:**
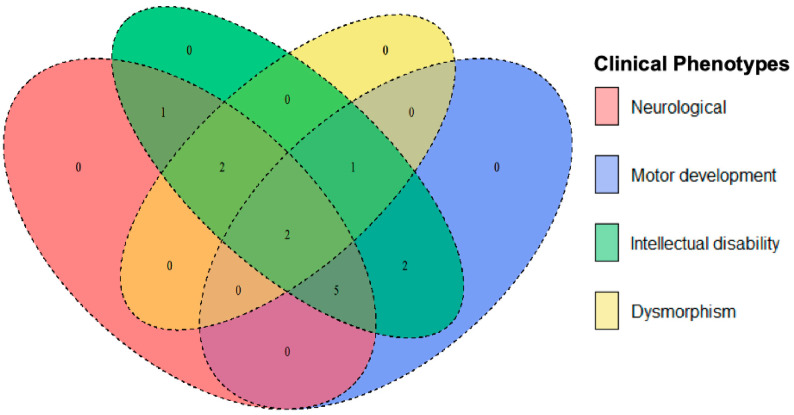
The Venn diagram visually represents the overlap of clinical phenotypes among different genetic conditions. **Orange**: neurological features (e.g., seizures, hypotonia, spasticity). **Blue**: motor development issues (e.g., loss of ambulation, fine motor impairment). **Green**: intellectual disability (mild to severe cognitive impairment). **Yellow**: dysmorphism (distinct facial or skeletal anomalies).

The Venn diagram illustrates the genetic overlap among major clinical phenotypes, including neurological features (seizures, hypotonia, and spasticity), motor development challenges (including loss of ambulation and fine motor impairment), ID, and dysmorphism (distinct facial or skeletal anomalies) (Figure 3, Table 3). This overlap suggests that genes influencing neurological function often contribute to both motor and cognitive dysfunction, which is consistent with the shared neurological pathways regulating both movement and cognition. Examples include *SCN2A*, *IQSEC2*, and *ADSL*, whose mutations are associated with seizures, motor delays, and severe intellectual disability. Likewise, *PLP1*, *NKX6-2*, and *ATP1A3* mutations are linked to spasticity, ataxia, and cognitive deficits.

Pathogenic variants in genes, such as *PGAP3*, *CHD3*, and *SMAD6*, can impair motor and cognitive performance without producing major neurological symptoms like epilepsy, suggesting that these deficits arise from developmental or structural abnormalities rather than from direct neurodegeneration.

Mutations associated with syndromic conditions often combine neurological and cognitive impairments with dysmorphic features. Loss-of-function variants in *NIPBL* cause Cornelia de Lange syndrome; missense variants in *ACTG1* lead to Baraitser-Winter syndrome; missense variants in *ATP1A3* underlie Snijders Blok-Campeau syndrome; and loss-of-function variants in *TCF4* result in Pitt-Hopkins syndrome. These syndromes usually present with both facial and skeletal anomalies, reflecting their broad developmental impacts. In contrast, loss-of-function variants in *SMAD6* are primarily associated with craniosynostosis, where skeletal malformations dominate, and neurological effects are limited.

### 3.4. Multidimensional and Pathway-Level Analysis of Genotype–Phenotype Associations

Given the absence of demographic bias, further multidimensional analyses were performed to investigate genotype–phenotype relationships and pathway-level clustering. Using the semi-quantitative clinical scoring system, numerical scores were assigned to major phenotypic domains for each patient carrying monogenic variants (Table 4).

This standardized approach enabled objective assessment of clinical severity across individuals and facilitated correlation and clustering analyses to delineate genotype–phenotype relationships. No consistent sex-related differences were observed across most clinical domains, indicating that disease severity and phenotypic distribution were generally comparable between males and females. Minor variability was noted in a few parameters (e.g., neurological, cognitive, and EEG domains), but none reached statistical significance after correction (*p* > 0.05, Mann–Whitney U test) (Supplementary Figure S4).

Similarly, no significant correlation between age and overall clinical severity was observed across most domains, suggesting that genotype rather than age progression predominantly determined disease expression. Mild trends were noted; for example, older patients tended to have slightly higher neurological and vision scores, whereas onset and ID scores decreased with age, potentially reflecting developmental compensation or diagnostic timing effects (Supplementary Figure S5).

**Figure 4:**
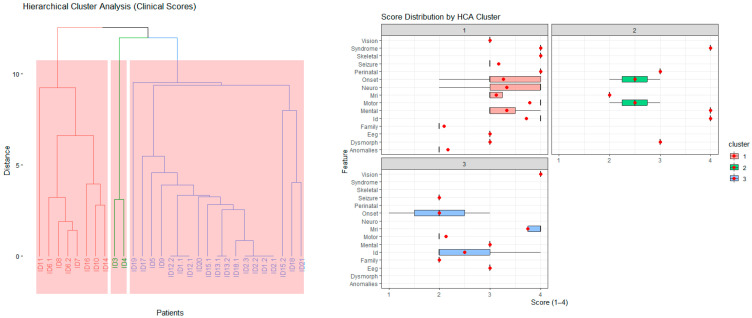
(**Left**) Hierarchical Cluster Analysis (HCA) of Clinical Severity Scores. Dendrogram representing hierarchical cluster analysis (HCA) based on standardized clinical severity scores across 15 domains. Each branch corresponds to an individual patient, and the height of linkage reflects Euclidean distance between multivariate score profiles. Three major clusters were identified, indicating distinct phenotypic patterns among patients. (**Right**) Distribution of Clinical Severity Scores Across HCA Clusters: Box plots showing the distribution of semi-quantitative clinical severity scores (1–4) for each phenotypic domain, stratified by hierarchical cluster group (1–3) derived from HCA. Each dot represents an individual patient score.

The HCA identified distinct patient subgroups with shared clinical characteristics. The left (red) cluster included individuals with early onset, severe multisystem involvement (e.g., neonatal onset, high neurodevelopmental and MRI severity scores), whereas the right (blue) cluster comprised patients with milder or later-onset phenotypes. These clusters indicate genotype–phenotype convergence and provide a framework for subsequent pathway-level and gene-based analyses (Figure 4, left).

Distinct clinical patterns emerged among the three HCA clusters. Cluster 1 (red) exhibited elevated scores across multiple domains (e.g., onset, neurological, cognitive, MRI, and seizure), reflecting early-onset, severe multisystem disease. Cluster 2 (green) displayed intermediate severity, particularly in neurodevelopmental and cognitive domains, representing moderate phenotypes. Cluster 3 (blue) showed relatively milder and more localized abnormalities, often dominated by motor and perinatal features. These distributions demonstrate that HCA effectively stratified patients into biologically meaningful subgroups with differing phenotypic severity profiles (Figure 4, right).

Hierarchical cluster analysis (HCA), based on Euclidean distances of standardized Z-scores across 15 clinical domains (e.g., age at onset, motor function, neurodevelopment, MRI findings, and seizures), stratified patients—not genes—into three distinct phenotypic groups (Figure 4). Each group represented a unique constellation of disease severity, onset, and system involvement. Notably, this patient-level clustering was derived exclusively from multidimensional clinical similarity, entirely independent of genotype. Gene and pathway annotations were subsequently incorporated post hoc to assess the biological coherence of the resulting patient clusters.

Group 1—Severe multisystem neurodevelopmental disorders (Transcriptional/RNA-processing phenotype): This group included patients carrying variants in *POLR1C, TCF4, HNRNPU, NIPBL,* and *ACTG1*. These patients exhibited the highest composite Z-scores (≥+2) across onset, neurological, motor, and MRI domains, corresponding to early-infantile onset, profound motor dysfunction, extensive cortical and white-matter abnormalities, and severe intellectual disability. Functionally, these genes are involved in transcriptional regulation, chromatin remodeling, and RNA-processing mechanisms, and their disruption leads to global developmental arrest. Representative examples include *POLR1C* (RNA polymerase dysfunction), *TCF4* (Pitt–Hopkins syndrome), and *HNRNPU* (epileptic encephalopathy). Thus, Group 1 represents the most severe, multisystem neurodevelopmental phenotype characterized by early developmental arrest and transcriptional dysregulation.

Group 2—Intermediate epilepsy/metabolic disorders (Ion-channel and neuronal-excitability phenotype): This group comprised patients harboring variants in *SCN2A, PAH, IQSEC2,* and *GNPAT*. These patients exhibited moderate overall severity, characterized by high seizure Z-scores (>+2), intermediate onset and neurological scores, and minimal systemic or dysmorphic involvement. The phenotype was dominated by neuronal excitability and metabolic dysfunction primarily affecting the central nervous system. Pathogenically, *SCN2A* variants alter voltage-gated sodium channel activity, leading to early infantile epileptic encephalopathy, whereas *PAH* and *GNPAT* variants disrupt amino acid and peroxisomal metabolism, respectively. Thus, Group 2 represents an intermediate neuro-motor severity profile driven predominantly by epileptic–metabolic mechanisms.

Group 3—Mild or focal neurodevelopmental presentations (Myelination/structural-signaling phenotype): This group comprised patients carrying variants in *NKX6-2, PLP1, PGAP3, SMAD6,* and *ATP1A3*. These patients exhibited the lowest composite Z-scores (<+1.5) and later onset, typically diagnosed after two years of age. Clinically, they presented with partial motor delay, mild intellectual disability, or leukodystrophy-like changes, occasionally accompanied by subtle visual or perinatal involvement. Functionally, these genes are implicated in myelination and intracellular signaling pathways—for example, *NKX6-2* (oligodendrocyte differentiation), *PLP1* (myelin formation), and *SMAD6* (TGF-β signaling modulation). Thus, Group 3 represents milder or more localized neurodevelopmental disorders with limited systemic impact and slower progression.

**Figure 5:**
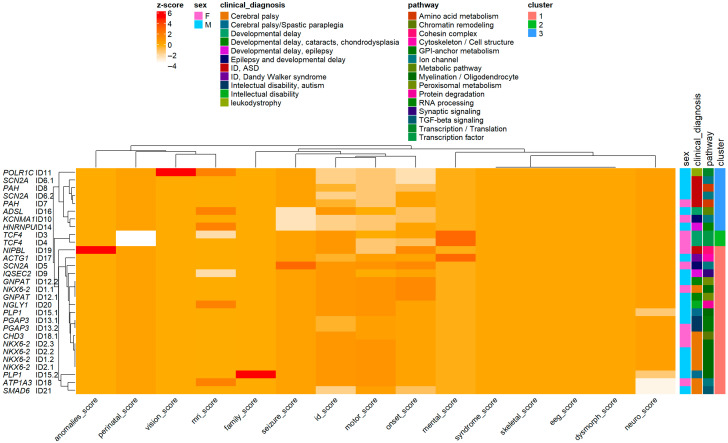
Integrated Heatmap of Clinical Severity, Pathway, and Diagnostic Features. Heatmap integrating z-scored clinical severity profiles (rows) and individual patient data (columns), annotated by cluster assignment, sex, clinical diagnosis, and molecular pathway. Hierarchical clustering was performed on both axes (Euclidean distance, Ward’s linkage) to visualize phenotypic co-variation and potential genotype–phenotype convergence. The color scale represents standardized z-scores of severity (from low [white] to high [red]), while side bars denote categorical annotations for each patient.

Although the HCA was based solely on Euclidean distances of clinical Z-scores, subsequent gene annotation revealed non-random biological clustering among patient groups. Pearson’s chi-squared test confirmed a significant association between gene distribution and phenotypic groups (χ^2^ = 54.566, df = 34, *p* = 0.0141), indicating that genes and pathways were not randomly distributed across clusters. Consistent with this, pathway-level enrichment analyses (Figure 5) (Supplementary Figures S6–S8) demonstrated distinct biological signatures: Group 1, transcriptional and RNA-processing pathways; Group 2, ion-channel and metabolic pathways; and Group 3, myelination and structural/signaling pathways. Together, these findings confirm that Euclidean-based patient clustering effectively captured the underlying genotype–phenotype architecture, reinforcing the biological validity and analytical robustness of the multidimensional HCA framework.

**Figure 6:**
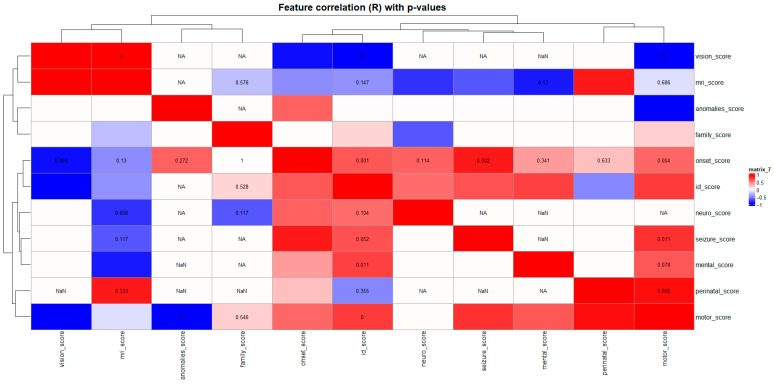
Correlation Matrix of Clinical Severity Domains (R with *p*-values). Heatmap illustrating pairwise Pearson correlation coefficients (R) among clinical severity domains, with overlaid *p*-values. Red shades indicate positive correlations, blue shades indicate negative correlations, and color intensity corresponds to the correlation strength. Hierarchical clustering was applied to both axes to identify co-varying clinical features.

The correlation matrix of clinical severity domains (Figure 6) revealed a well-structured internal architecture, reflecting strong interrelationships among neurodevelopmental aspects of the phenotype. ID, neurological, motor, and mental domains were strongly correlated (*R* ≈ 0.6–0.8, *p* < 0.05), indicating that the degree of motor and behavioral impairments parallels cognitive dysfunction. This pattern highlights a coherent neurocognitive cluster in which central nervous system dysfunction drives both intellectual and behavioral outcomes.

A significant correlation was also found between perinatal and onset scores (*R* ≈ 0.6, *p* < 0.05), suggesting that perinatal complications may accelerate disease onset or exacerbate symptom severity. In contrast, vision and MRI domains showed weak or non-significant correlations with other traits, indicating genotype-specific or structurally localized effects rather than generalized markers of severity.

Collectively, the correlation map delineates a multidimensional yet internally structured clinical phenotype architecture. While neurodevelopmental domains exhibited strong interrelationships, sensory and imaging parameters were more variable. These findings validate the semi-quantitative clinical scoring system as a biologically meaningful tool for subsequent hierarchical clustering and multidimensional pathway integration analyses.

### 3.5. Copy Number Variants in Children with ID

Ten children with ID harbored CNVs. Among them, two had only duplications (CNV1, CNV2), six had only deletions (CNV3-8), and two had both duplications and deletions (CNV9, CNV10). CNV sizes ranged from 1.3 Mb to 19.1 Mb. The 6.2 Mb duplication on 16p detected in proband CNV1 was a de novo variant, whereas the 19.1 Mb duplication on 6q detected in proband CNV2 was derived from a paternal balanced chromosomal translocation t(6;20)(q25;p13). In addition, two unrelated children (CNV4, CNV7) carried a de novo deletion del(7)(q11.23), which is linked to Williams-Beuren syndrome.

**Figure 7:**
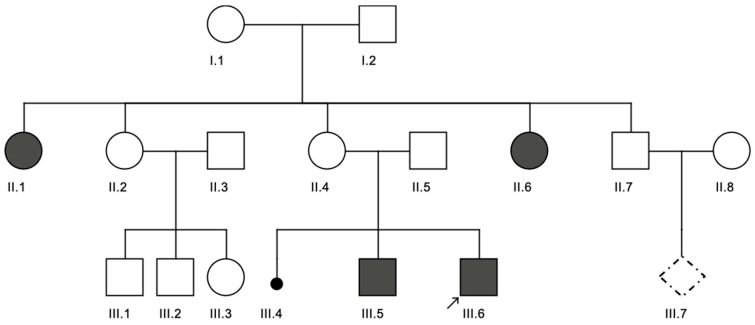
The three-generation pedigree of proband CNV8 (III.6, index). Data highlight that two aunts (II.1 and II.6) and two siblings (III.5 and III.6) display intellectual disabilities along with distinct facial features, such as ptosis. Notably, both aunt II.1 and sibling III.6 possess the del(9)(q31.1-q31.3) variant, while their parents (II.2 & II.3; I.1 & I.2) show normal CNV and karyotype results. This indicates the possibility of a cryptic balanced rearrangement among other healthy members. Therefore, a prenatal diagnostic test was performed in the fetus (III.7) and no CNV or karyotype abnormality was detected. The black-filled symbols indicate affected individuals, and the arrows point to the patients who were clinically examined.

Interestingly, two patients exhibited both a terminal deletion and a terminal duplication despite normal parental karyotypes and CNV sequencing results. The first case (CNV9) involved an eight-year-old girl with a 6.7 Mb terminal duplication on 10p and a 6.9 Mb terminal deletion on 18q, suggesting either a spontaneous unbalanced chromosomal translocation or a cryptic parental chromosomal rearrangement. The second case (CNV10) was a four-year-old boy with a 15 Mb duplication at 2q36.3–q37.3 and a 2.5 Mb deletion at 4q35.2. Furthermore, an eight-year-old boy (CNV8) carried a 10.2 Mb deletion on chromosome 9q and had a family history of multiple affected relatives with the same CNV, despite his parents having normal karyotypes and CNV sequencing results (Figure 7).

Phenotypic evaluation of the CNV-positive cohort revealed that five of the ten patients displayed prenatal abnormalities detectable by ultrasound, and all exhibited dysmorphic facial features. Three patients had congenital heart defects, and three male patients presented with testicular abnormalities. Regarding cognitive outcomes, six patients had severe-to-profound ID, one had mild ID, and the remainder had moderate ID. In terms of motor development, three children were unable to walk, whereas the others experienced mild-to-moderate motor delays (Table 5 and Table 6).

### 3.6. Unconfirmed Cases

Seven cases with monogenic variants remained inconclusive. Notably, three of these patients carried biallelic variants in genes associated with autosomal recessive disorders, classified as variants of uncertain significance (VUS) but with high predicted pathogenicity according to ACMG/AMP/ClinGen SVI guidelines.

The first case (ID22) was an eight-year-old girl carrying an in-cis compound heterozygous variant in the *OSGEP* gene: a paternal c.280C>T (p.Arg94Cys) and a maternal c.143A>G (p.His48Arg) variant. Both missense variants were classified as VUS. Clinically, the patient exhibited severe ID, nephrotic syndrome, loss of ambulation, and loss of speech, features consistent with the clinical features of Galloway–Mowat syndrome type 3 (GAMOS3) [30]. Her older brother with similar clinical manifestations had passed away without undergoing genetic testing.

The second case (ID23) was a 16-year-old boy presenting with speech delay, moderate ID, normal motor development, and attention-deficit/hyperactivity disorder (ADHD). He carried an in-trans compound heterozygous variants in the *HEPHL1* gene: a maternal c.2857C>T (p.Arg953Ter) and a paternal c.84G>A (p.Thr28=) variant. The c.2857C>T variant introduces a premature termination codon (PTC) and is classified as pathogenic, whereas the c.84G>A variant, located outside the splice region and not predicted to alter splicing, is classified as VUS despite occurring in trans with a pathogenic allele.

The third case (ID24) involved an 11-year-old girl with profound intellectual disability, loss of ambulation, and hypotonia. Her deceased older brother exhibited similar symptoms but had not undergone genetic testing. The patient carried a homozygous c.1066C>T variant in the *PRUNE1* gene, inherited from both parents. Biallelic pathogenic variants in *PRUNE1* have been known to cause neurodevelopmental disorder with microcephaly, hypotonia, and brain abnormalities [31]. The identified variant, a null mutation in the last exon, is not predicted to undergo nonsense-mediated decay (NMD) but likely results in truncation of more than 10% of the transcript. Its extremely low frequency in the gnomAD population supports classification as a VUS with high pathogenic potential. Prenatal diagnostic testing performed during the mother’s subsequent pregnancy did not detect the *PRUNE1* variant, and the newborn, now one year old, exhibits normal psychomotor development.

The recurrent *MTSS2*:c.1790C>T variant was found in two unrelated cases. Although previously reported as likely pathogenic [32], its high allele frequency in East Asian populations (gnomAD genome frequency: 0.002327) suggests reduced pathogenicity. Moreover, this variant was recently listed among genes excluded from ACMG prenatal screening for genetic disorder with high allele frequency (>1%) in VN1K genome database, a population scale multiomics and phenomics resource for the Vietnamese population) [33]. In both families, the variant was inherited from a phenotypically normal parent, supporting its reclassification as a VUS with low pathogenic potential.

Additionally, one child (ID26) carried a loss-of-function (LOF) variant in *ATAD3A*, associated with a known genetic disorder, but inherited from an unaffected mother. Another patient (ID25) had an LOF variant in *COL4A1*, inherited from an unaffected father. Due to the variable penetrance and expressivity of these genes, both variants were ultimately classified as VUS.

## 4. Discussion

This study highlights the genetic complexity of ID, demonstrating that both SNVs and CNVs significantly contribute to the disease spectrum. Our data revealed a male predominance, with males affected at approximately twice the frequency of females, consistent with previous reports [34,35]. Notably, SNV-associated ID cases were generally diagnosed later than CNV-associated cases, suggesting that early genetic screening could facilitate timely diagnosis and improve disease management. Prior international studies have similarly shown that early implementation of genomic testing—particularly exome or genome sequencing—shortens the diagnostic odyssey and enhances clinical care for patients with neurodevelopmental disorders [3].

WES and CES were the primary tools used to identify both SNVs and CNVs in coding regions, representing a practical, dual-purpose approach in resource-limited settings [34]. However, CNV analysis based on WES/CES data cannot reliably detect balanced structural variants, mosaicism, and small CNVs [36], highlighting the need for complementary testing in unresolved cases such as CNV8. Importantly, several CNV-positive patients exhibited prenatal abnormalities detectable by ultrasound but remained undiagnosed before birth, underscoring the value of incorporating genomic testing into routine prenatal diagnostics [32]. Furthermore, trio-based sequencing, which was not consistently applied in this cohort, could improve diagnostic accuracy by identifying de novo mutations—a key indicator of pathogenicity in dominant disorders [37].

From a genetic perspective, this study uncovered recurring mutations in genes, such as *NKX6-2*, *PAH*, and *SCN2A*, where frameshift and nonsense mutations were associated with pronounced phenotypic effects. The high frequency of missense mutations underscores the need for functional studies to assess their pathogenic potential. Additionally, splice-site mutations, which can result in exon skipping or aberrant protein synthesis, point to diverse molecular mechanisms underlying ID. These findings emphasize the importance of integrating genetic and clinical data to refine variant interpretation and enhance diagnostic precision.

Phenotypic variability among patients carrying mutations in the same gene was also evident. For example, *SCN2A* variants produced a broad clinical spectrum ranging from epilepsy to severe neurodevelopmental disorders, reflecting variable expressivity and genotype-specific functional effects [38]. While many genes contribute to the heterogeneity of ID, isolated cases remain relatively rare. *PAH* mutations, for instance, mainly result in PKU, which, if left untreated, leads to neurological impairment without significant dysmorphism or motor difficulties. This highlights the critical importance of early metabolic screening and intervention, as timely dietary and metabolic management can prevent or substantially reduce neurological deterioration [39]. Clinically, early genetic testing is particularly valuable in patients presenting with combined neurological, motor, and intellectual impairments. The presence of dysmorphism alongside neurodevelopmental delays should prompt consideration of syndromic disorders such as Cornelia de Lange, Pitt-Hopkins, and Baraitser-Winter syndromes.

For cases with VUS, some patients exhibited biallelic variants, consistent with the suspected diagnosis and family history, yet these did not meet the current ACMG/AMP/Clingen criteria for pathogenicity [40,41]. In such instances, functional validation studies or segregation analysis in first-degree relatives are recommended to strengthen variant interpretation. Furthermore, for families harboring potentially deleterious variants, prenatal or preimplantation genetic testing should be considered to mitigate recurrence risk in future pregnancies [42]. In cases where a deleterious variant was detected in both patient and a healthy parent, (e.g., patient ID25 with a heterozygous *COL4A1* variant) variable age of onset and incomplete penetrance warrant ongoing clinical surveillance and functional validation [43].

Visual tools, such as Venn diagrams, proved valuable for integrating genetic and phenotypic data, enabling clear visualization of overlapping domains, including intellectual disability, motor deficits, and dysmorphism. Expanding this framework to incorporate additional clinical subcategories—such as severity of ID or epilepsy subtypes—could further enhance diagnostic precision and support personalized management strategies.

Collectively, these findings underscore the importance of early genetic screening, rigorous variant classification, and the use of complementary approaches, including long-read sequencing and functional assays, to resolve inconclusive cases. This study employed an integrative, multidimensional analytic framework to characterize genotype–phenotype relationships in patients with ID and ASD. By combining semi-quantitative clinical scoring, correlation mapping, hierarchical cluster analysis, and pathway-level integration, we delineated the internal structure of phenotypic heterogeneity and its molecular correlates.

Our results demonstrated that the distribution of genetic diagnostic yield was not significantly influenced by sex or age, supporting the biological consistency of the analyzed cohort. The semi-quantitative clinical scoring system, encompassing 15 domains—including developmental, neurological, structural, and systemic features—provided a harmonized and granular representation of disease burden. This approach, consistent with prior multidimensional frameworks [19,20,21,22,23,24,25,26], enabled systematic evaluation of phenotypic variation across functional systems.

The correlation heatmap revealed a coherent internal architecture within the dataset. Strong positive correlations among neurodevelopmental domains—intellectual disability, motor, mental, and neurological features—defined a unified neurocognitive functional cluster, indicating that neuronal dysfunction underlies both cognitive and motor outcomes. The observed association between perinatal complications and early disease onset underscores the modifying role of early-life biological stress on phenotypic expression. In contrast, sensory (vision) and neuroimaging (MRI) domains exhibited weak correlations with other traits, suggesting modular or genotype-specific effects rather than generalized severity.

HCA stratified patients into three major clusters, each reflecting distinct severity profiles and phenotypic compositions. Group 1 encompassed individuals with the most severe neurodevelopmental impairment and multiple coexisting systemic abnormalities. Group 2 comprised patients with moderate delays and partial functional preservation, whereas Group 3 primarily included milder cases with localized or isolated deficits. This stratification validates the semi-quantitative scoring model and demonstrates its capacity to recapitulate clinically meaningful subgroups.

Integration with pathway-level data reinforced these clinical groups. The combined heatmap of Z-scored phenotypic traits and annotated molecular pathways revealed that patients in the severe cluster were enriched for variants affecting ion channel function, myelination, metabolic processes, and synaptic signaling—mechanisms central to neuronal excitability and brain development. In contrast, milder clusters were more frequently associated with transcriptional or RNA-processing pathways. This gradient, from metabolic–synaptic to transcriptional mechanisms, mirrors the transition from early developmental to regulatory dysfunction, highlighting the biological coherence of the clustered phenotypes.

Collectively, these findings underscore the utility of multidimensional, semi-quantitative, and unsupervised methods in elucidating clinical–genetic complexity. By leveraging inter-domain correlations and hierarchical structure, these approaches enable the identification of phenotypic convergence patterns that univariate analyses may overlook. Moreover, the observed modularity supports the use of pathway-based modeling to refine variant interpretation and guide future functional studies.

Our study is consistent with recent Vietnamese and international research applying hierarchical clustering, correlation networks, and machine-learning–based dimensionality reduction to elucidate genotype–phenotype interactions in complex genetic disorders, including hepatocellular carcinoma, ischemic stroke, and viral infections. The reproducibility of these data-driven approaches across diverse biological contexts underscores their robustness and translational relevance in precision genomics.

The study sample size was limited and derived from a single center; however, the cohort included patients from diverse ethnic and geographic backgrounds across Vietnam. Variants located in non-coding regions, cryptic balanced chromosomal abnormalities, and other genomic alterations undetectable by ES or CES may have been missed. In certain cases (e.g., CNV8), the familial origin of the mutation could not be determined. Future studies should focus on expanding cohort size and incorporating patients from multiple centers. The application of whole-genome sequencing (WGS) and long-read sequencing technologies is recommended to broaden the genomic landscape assessed and enhance the detection of complex or previously undetectable variants. These strategies will ultimately improve diagnostic accuracy and enable more personalized treatment approaches for neurodevelopmental disorders.

In summary, this multidimensional framework delineates clinically coherent subgroups among ID/ASD patients and bridges the gap between genetic mechanisms and phenotypic outcomes. Future extensions incorporating trio-based sequencing, methylation profiling, and longitudinal data will further refine genotype–phenotype mapping, advancing precision diagnosis and personalized management in neurodevelopmental genetics.

## Figures and Tables

**Table 1:** Clinical scoring domains and criteria (scores 1–4).

Domain	Score 1 (Mild/Late)	Score 2 (Moderate)	Score 3 (Early/Severe)	Score 4 (Severe/Neonatal/Multisystemic)	NA/Normal
**1** **. Onset characteristics**	≥2–3 years, mild onset (e.g., speech delay 3 y, hand tremor 5 y)	12–24 months, walking/speech delay	4–12 months, early motor delay (head lag 4–7 mo, nystagmus infantile)	Neonatal–3 mo, severe multisystem onset (nystagmus neonatal, seizures, dysmorphy)	Normal/not recorded
**2. Perinatal history**	Unremarkable pregnancy/delivery	Mild–moderate risk (e.g., maternal illness, late preterm)	Significant abnormalities (e.g., oligohydramnios, abnormal fetal ultrasound)	Major complications (e.g., asphyxia, intracranial bleed, hydrops)	Normal/not documented
**3. Vision/Eyes**	Minor or transient issues	Mild anomaly (e.g., strabismus)	Persistent nystagmus or abnormal eye movements	Severe visual loss, optic atrophy	Normal/not done
**4. Neurological/Neuromuscular**	Subtle neurological signs	Mild hypertonia/clumsiness	Focal spasticity or dystonia	Severe spastic tetraplegia or hypotonia	Normal/not done
**5. Seizures (onset)**	None or doubtful	Febrile or >12 mo, controlled	<12 mo (non-febrile, recurrent)	Neonatal–3 mo, frequent or intractable	No/N/A
**6. EEG (Electroencephalography)**	Near normal	Mild nonspecific slowing	Clear focal/generalized spikes	Severe patterns (hypsarrhythmia, burst-suppression)	Normal/not done
**7. Brain MRI**	Subtle/equivocal	Mild lesions (nonspecific, mild ventriculomegaly)	Moderate abnormalities (delayed myelination, degeneration)	Severe/structural malformation (hydrocephalus, vanishing WM)	Normal/not done
**8. Mental/Behavioral disorders**	Mild symptoms	Suggestive signs (attention/motor coordination problems)	Formal ASD/ADHD diagnosis, functional impact	Severe behavior disturbance, ASD with aggression	No/N/A
**9. Motor development**	Mild delay/clumsy	Moderate delay, partial support	Severe delay, cannot walk without help	Loss of ambulation/never walked	Normal
**10. Intellectual Disability**	Borderline/learning difficulty	Mild	Moderate	Severe/profound	Normal/not assessed
**11. Dysmorphism**	Very mild/doubtful	Mild, not typical	Clear facial or systemic dysmorphism	Syndromic, multi-systemic features	None
**12. Skeletal system**	Very mild/suspected	Mild deformity	Moderate deformity (localized contracture)	Severe/multiple deformities (talipes, contractures, atrophy)	Normal
**13. Accompanying anomalies/conditions**	Minor/nonsignificant	Mild recurrent (e.g., respiratory illness)	Single-organ significant (e.g., vesicoureteral reflux)	Multi-organ involvement (e.g., VUR + craniosynostosis)	Blank
**14. Family history**	Negative	Suspected distant	First-degree affected	Multiple relatives/clear inheritance	Unknown
**15. Syndrome/Non-syndrome**	No syndromic features	Focal phenotype only (ID/ASD)	Suspected syndrome (dysmorphism + EEG/MRI pattern)	Confirmed syndrome (e.g., Pitt–Hopkins, Cornelia de Lange)	Blank

NA: not applicable.

**Table 2:** Genetic Impact on Clinical Phenotypes.

Gene	Number of Cases	Main Clinical Phenotype	Neurological Features	Motor Development	Intellectual Disability	Dysmorphism	Associated Syndrome
*IQSEC2*	1	Developmental delay, epilepsy	Seizures (1 year old)	Severe delay	Severe delay	No	No
*KCNN4*	1	Epilepsy, developmental delay	Seizures (13 months)	Mild delay	Mild delay	No	No
*POLRIC*	1	Leukodystrophy	No seizures	Mild delay (hand tremor)	Mild delay	No	No
*GNPAT*	2	Developmental delay, skeletal anomalies	Hypotonia	Loss of ambulation	Severe delay	Joint contractures	No
*PGAP3*	2	Developmental delay, autism	Unclear	Mild delay (fine motor impairment)	Moderate delay	Jaw anomalies	No
*HNRNPU*	1	Developmental delay, epilepsy	Febrile seizures	Mild delay	Mild delay	No	No
*PLP1*	2	Cerebral palsy, spastic paraplegia	Spasticity, muscle atrophy	Loss of ambulation	Severe delay	No	No
*ADSL*	1	Developmental delay	Seizures	Mild delay	Severe delay	No	No
*ACTG1*	1	Dandy-Walker syndrome	No neurological issues	Fine motor impairment	Moderate delay	Widely spaced eyes, slanted eyes	Baraister-Winter
*ATP1A3*	1	Cerebral palsy	Hypotonia, hemiplegia	Loss of ambulation	Severe delay	Frontal bossing, round face	Snijders Blok-Campeau syndrome
*CHD3*	1	Cerebral palsy	Unclear	Loss of ambulation	Severe delay	Frontal bossing, round face	No
*NIPBL*	1	Autism, developmental delay	Unclear	Moderate delay	Moderate delay	Vesicoureteral reflux	Cornelia de Lange
*NGLY1*	1	Intellectual disability	Hypotonia	Loss of ambulation	Severe delay	Cryptorchidism	No
*SMAD6*	1	Cerebral palsy, skull anomalies	Hypotonia	Gross and fine motor delay	Mild delay (speech delay)	Premature craniosynostosis, hip dislocation	No
*NKX6-2*	5	Hereditary spastic paraplegia	Spasticity, hyperreflexia	Loss of ambulation	Moderate delay	No	No
*TCF4*	2	Global developmental delay	Seizures, intellectual disability	Hypotonia	Severe delay	Distinct facial features	Pitt-Hopkins
*PAH*	2	Autism, Intellectual disability	Intellectual disability (if untreated)	Normal	Severe delay (if untreated)	Microcephaly	No
*SCN2A*	3	Epileptic encephalopathy	Infantile seizures	Hypotonia	Severe delay	No	No

**Table 3:** Summarizes the Overlap of Genetic Conditions across the Four Major Clinical Phenotypes: Neurological Features, Motor Development Issues, Intellectual Disability, and Dysmorphism.

Overlap Category	Genes Involved	Key Clinical Insights
Neurological + Motor + Intellectual Disability	*IQSEC2, SCN2A, ADSL, PLP1, NKX6-2, ATP1A3*	Strong overlap suggests shared neurological pathways affecting motor function and cognition.
Motor + Intellectual Disability (No Neurological Signs)	*PGAP3, CHD3, SMAD6*	Likely structural or developmental causes rather than primary neurodegeneration.
Neurological + Intellectual Disability (No Motor Impairment)	*TCF4, PAH*	TCF4 (Pitt-Hopkins) → severe ID and epilepsy; PAH (Phenylketonuria) → treatable if diagnosed early.
Dysmorphism + Neurological + Intellectual Disability	*NIPBL, ACTG1, ATP1A3, TCF4*	Found in syndromic conditions (e.g., Cornelia de Lange, Pitt-Hopkins, Baraister-Winter).
Dysmorphism + Motor + Intellectual Disability	*SMAD6, ACTG1*	Craniofacial or skeletal anomalies paired with motor and cognitive impairments.
Neurological Features Only	*PAH*	Phenylketonuria leads to neurological issues but no motor or dysmorphic features.
Dysmorphism Only	None	No genes exclusively cause dysmorphism without cognitive or motor impairment.

**Table 4:** Semi-scoring in Monogenic variants phenotypes.

Id	Age	Sex	Gene	Clinical Diagnosis	Onset	Perinatal	Vision	Neuro	Seizure	Eeg	Mri	Mental	Motor	Id	Dysmorph	Skeletal	Anomalies	Family	Syndrome
ID1.1	2014	F	NKX6-2	Cerebral palsy	4		3	4					4	4			2	2	
ID1.2	2009	M	NKX6-2	Cerebral palsy			3	4					4	4			2	2	
ID2.1	2019	M	NKX6-2	Cerebral palsy	3		3	4	3	3	3		4	4			2	2	
ID2.2	2013	M	NKX6-2	Cerebral palsy	3		3	4	3	3	3		4	4			2	2	
ID2.3	2011	F	NKX6-2	Cerebral palsy	3		3	4	3	3	3		4	4			2	2	
ID3	2019	F	TCF4	Developmental delay	3	3					2	4	3	4					4
ID4	2016	F	TCF4	Developmental delay	2	3						4	2	4	3				4
ID5	2014	F	SCN2A	Epilepsy and developmental delay	4				4				4	4					
ID6.1	2010	M	SCN2A	ID, ASD	1							3	2	2				2	
ID6.2	2014	M	SCN2A	ID, ASD								3	2	2				2	
ID7	2013	F	PAH	ID, ASD	3						3	3	2	3					
ID8	2015	M	PAH	ID, ASD	2							3	2	3					
ID9	2018	M	IQSEC2	Developmental delay, epilepsy	3				3	3	2		3	4					
ID10	2018	F	KCNMA1	Epilepsy and developmental delay	2				2	3			2	2					
ID11	2002	M	POLR1C	leukodystrophy	1		4				4	3	2	2					
ID12.1	2010	M	GNPAT	Developmental delay, cataracts, chondrodysplasia	4								4	4		4		2	
ID12.2	2014	M	GNPAT	Developmental delay, cataracts, chondrodysplasia	4								4	4		4		2	
ID13.1	2020	M	PGAP3	Intelectual disability, autism								3		3				2	
ID13.2	2014	F	PGAP3	Intelectual disability, autism								3		3				2	
ID14	2019	M	HNRNPU	Developmental delay, epilepsy	3				2	3	4	3	2	2				2	
ID15.1	2015	M	PLP1	Cerebral palsy/Spastic paraplegia	3	4	3	3			3		4	4		4		2	
ID15.2	2018	M	PLP1	Cerebral palsy/Spastic paraplegia	3		3	3			3		4	4		4		3	
ID16	2016	M	ADSL	Developmental delay	2				2	3	4		3	4				2	
ID17	2015	M	ACTG1	ID, Dandy Walker syndrome	3	4						4		3	3				4
ID18	2019	F	ATP1A3	Cerebral palsy	3	4		2	3		4		4	4	3				
ID18.1	2019	F	CHD3	Cerebral palsy															4
ID19	2018	F	NIPBL	ID, ASD	4								2	4			3		4
ID20	2020	M	NGLY1	Intellectual disability	3						4		4	4					
ID21	2018	M	SMAD6	Cerebral palsy	2	4		2						2					

**Table 5:** Overall CNV mutation.

ID	Age	Sex	Type	Chr	CNV	Location	Length	Genes	CNV Syndrome Variants	PMID	Class	Parental Origin
CNV1	2019	F	dup	16	dup(16)(p13.3)	50,000_6,249,999	6,2 Mb	265	16p13.3 duplications	36586055	P	De novo
CNV2	2018	M	dup	6	dup(6)(q25.1-q27)	151,478,865_170,590,912	19.1 Mb	177	No		LP	Pat (t(6;20)(q.25;p.13)
CNV3	2018	M	del	6	del(6)(q12-q14.1)	67,390,107_76,290,283	8.9 Mb	87			LP	De novo
			del	15	del(15)(q21.1-q21.3)	48,907,803_54,907,802	6.0 Mb	67			LP	
CNV4	2007	F	del	7	del(7)(q11.23)	73,285,998_74,585,671	1.3 Mb	34	Williams-Beuren Syndrome	34140529	P	De novo
CNV5	2017	M	del	5	del(5)(q14.2-q14.3)	82,604,181_90,004,183	7.4 Mb	54	5q14.3 microdeletion syndrome	26161356		De novo
CNV6	2018	M	del	14	del(14)(q32.2)	98,433,663_100,733,663	2.3 Mb	28		18197200	P	De novo
CNV7	2016	M	del	7	del(7)(q11.23)	73,285,998_74,585,671	1.3 Mb	34	Williams-Beuren Syndrome	34140529	P	De novo
CNV8	2017	M	del	9	del(9)(q31.1-q31.3)	101,537,718_111,737,720	10.2 Mb	100		24376033, 36461789	P	Uncertain
CNV9	2015	F	dup	10	dup(10)(q15.3- p14)	54,060_6,758,038	6.7 Mb	84			P	Uncertain
			del	18	del(18)(q22.3-q23)	73,332,765_80,219,365	6.9 Mb	54		17504501	P	
CNV10	2021	M	dup	2	dup(2)(q36.3-q37.3)	227,035,284_242,057,849	15.0 Mb	254			LP	Uncertain
			del	4	del(4)(q35.2)	187,378,846_189,878,845	2.5 Mb	18		22127048	VUS	

dup: duplication; del: deletion; F: female; M: male; Chr: Chromosome; Pat: Paternal.

**Table 6:** Clinical and Genetic Characteristics of Patients with CNV.

ID	Age	Sex	CNV	Perinatal History	Clinical Manifestation	Motor Development	Cognitive Development	Family History
CNV1	2019	F	dup(16)(p13.3)	Normal	Ptosis, hearing impairment, nasolacrimal duct obstruction	Moderate delay	Severe delay	No
CNV2	2018	M	dup(6)(q25.1-q27)	Increased nuchal translucency	Noonan- like Dysmorphia (neck webbing, low-set ears, down-slanting palpebral fissures, cryptorchidism, X-shaped legs, abnormal toes	Mild delay	Moderate delay	Yes (Mother had a pregnancy termination because of increased nuchal fold, hydrops fetalis)
CNV3	2018	M	del(6)(q12-q14.1)	Clef palate, colon obstruction	Clef palate, colon obstruction (surgery done), scoliosis, talipes equinovarus	Loss of ambulance	Sever delay	No
			del(15)(q21.1-q21.3)					
CNV4	2007	F	del(7)(q11.23)	Normal	Facial dysmorphy, congenital tricuspid regurgitation	Mild delay	Mild delay	No
CNV5	2017	M	del(5)(q14.2-q14.3)	Normal	Ventricular septal defect, cryptorchidism, seizures, MRI: white matter vanishing, cerebral atrophy	Loss of ambulance	Sever delay	No
CNV6	2018	M	del(14)(q32.2)	Normal	Facial dysmorphia, single transverse palmar crease, ventriculomegaly	Mild delay	Moderate delay	No
CNV7	2016	M	del(7)(q11.23)	Intrauterine growth restriction	Facial dysmorphia, aggressive behavior	Mild delay	Mild delay	No
CNV8	2017	M	del(9)(q31.1-q31.3)	Normal	Facial dysmorphia, ptosis)	Mild delay	Moderate delay	No
CNV9	2015	F	dup(10)(q15.3- p14)	Normal	Congenital tricuspid regurgitation, short stature	Mild delay	Moderate delay	No
			del(18)(q22.3-q23)					
CNV10	2021	M	dup(2)(q36.3-q37.3)	Intrauterine growth restriction	Nystagmus, abnormal eyes movement, hypotonia, seizures, facial dysmorphia, cryptorchidism	Loss of ambulation	Sever delay	Yes
			del(4)(q35.2)					

dup: duplication; del: deletion.

## Data Availability

The data sets used or analyzed during the current study are available from the corresponding author upon reasonable request, in compliance with institutional and ethical regulations. Due to privacy protection and ethical considerations, personal information or data that could identify individual patients cannot be shared.

## Supplementary Materials

The following supporting information can be downloaded at: https://www.mdpi.com/article/10.3390/diagnostics15222821/s1, Figure S1. Positive rate by sex among patients tested with WES/CES; Figure S2. Comparison of age between patients with positive and negative genetic findings; Figure S3. Age distribution by genetic test result; Figure S4. Distribution of clinical scores by sex; Figure S5. Relationship between age and clinical severity scores; Figure S6. Frequency of genes across HCA-derived clusters; Figure S7. Pearson’s Chi-squared test revealed a statistically significant association between gene distribution and hierarchical clusters (χ^2^ = 54.566, df = 34, *p* = 0.0141), indicating that gene occurrences were not randomly distributed across clusters. This finding supports the biological validity of the clustering, with specific genes (e.g., NKX6-2, PLP1, PGAP3) concentrated within transcriptional/myelination-related clusters, while others (e.g., SCN2A, PAH) grouped within ion-channel or metabolic pathways; Figure S8. Pathway-level distribution across HCA-derived clusters; Table S1. Monogenic variants; Table S2. Monogenic variants phenotypes; Table S3. Sum up the result of 75 cases.

## Abbreviations

The following abbreviations are used in this manuscript:

ID	Intellectual Disability
CNVs	Copy Number Variants
SNVs	Single-Nucleotide Variants
WES	Whole-Exome Sequencing
CES	Clinical Exome Sequencing
CMA	Chromosomal Microarray Analysis
NGS	Next-Generation Sequencing
ASD	Autism Spectrum Disorder
VUS	Variants of Unknown Clinical Significance
ClinVar	Clinical Variation database
OMIM^®^	Online Mendelian Inheritance in Man data
ACMG	American College of Medical Genetics and Genomics
AMP	Association for Molecular Pathology
ClinGen	Clinical Genome Resource
NCBI	National Center for Biotechnology Information
UCSC	University of California—Santa Cruz
IDT	Integrated DNA Technologies
PCR	Polymerase Chain Reaction
PKU	Phenylketonuria
SVI	Sequence Variant Interpretation

## References

[1] American Psychiatric Association (2013). Diagnostic and Statistical Manual of Mental Disorders.

[2] World Health Organization (1992). The ICD-10 Classification of Mental and Behavioural Disorders: Clinical Descriptions and Diagnostic Guidelines.

[3] Srivastava S., Love-Nichols J.A., Dies K.A., Ledbetter D.H., Martin C.L., Chung W.K., Firth H.V., Frazier T., Hansen R.L., Prock L. (2019). Meta-analysis and multidisciplinary consensus statement: Exome sequencing is a first-tier clinical diagnostic test for individuals with neurodevelopmental disorders. Genet. Med..

[4] Schalock R., Luckasson R., Tassé M. (2019). The contemporary view of intellectual and developmental disabilities: Implications for psychologists. J. Intellect. Disabil. Res..

[5] Maenner M.J., Shaw K.A., Bakian A.V., Bilder D.A., Durkin M.S., Esler A., Furnier S.M., Hallas L., Hall-Lande J., Hudson A. (2021). Prevalence of autism spectrum disorder among children aged 8 years—Autism and Developmental Disabilities Monitoring Network, 11 sites, United States, 2018. MMWR Surveill. Summ..

[6] Li M., Fallin M.D., Riley A., Landa R., Walker S.O., Silverstein M., Caruso D., Pearson C., Kiang S., Dahm J.L. (2016). The Association of Maternal Obesity and Diabetes With Autism and Other Developmental Disabilities. Pediatrics.

[7] Huang J., Zhu T., Qu Y., Mu D. (2016). Perinatal and Neonatal Risk Factors for Intellectual Disability: A Systemic Review and Meta-Analysis. PLoS ONE.

[8] Pierrat V., Marchand-Martin L., Arnaud C., Kaminski M., Resche-Rigon M., Lebeaux C., Bodeau-Livinec F., Morgan A.S., Goffinet F., Marret S. (2017). Neurodevelopmental outcome at 2 years for preterm children born at 22 to 34 weeks’ gestation in France in 2011: EPIPAGE-2 cohort study. BMJ.

[9] Camfield P.R., Bahi-Buisson N., Trinka E. (2014). Transition issues for children with diffuse cortical malformations, multifocal postnatal lesions, (infectious and traumatic) and Lennox-Gastaut and similar syndromes. Epilepsia.

[10] Shevell M., Ashwal S., Donley D., Flint J., Gingold M., Hirtz D., Majnemer A., Noetzel M., Sheth R.D. (2003). Practice parameter: Evaluation of the child with global developmental delay: Report of the Quality Standards Subcommittee of the American Academy of Neurology and the Practice Committee of the Child Neurology Society. Neurology.

[11] Vissers L.E.L.M., Gilissen C., Veltman J.A. (2015). Genetic Studies in Intellectual Disability and Related Disorders. Nat. Rev. Genet..

[12] Chiurazzi P., Pirozzi F. (2016). Advances in understanding–genetic basis of intellectual disability. F1000Research.

[13] Maulik P.K., Mascarenhas M.N., Mathers C.D., Dua T., Saxena S. (2011). Prevalence of intellectual disability: A meta-analysis of population-based studies. Res. Dev. Disabil..

[14] Miller D.T., Adam M.P., Aradhya S., Biesecker L.G., Brothman A.R., Carter N.P., Church D.M., Crolla J.A., Eichler E.E., Epstein C.J. (2010). Consensus Statement: Chromosomal Microarray Is a First-Tier Clinical Diagnostic Test for Individuals with Developmental Disabilities or Congenital Anomalies. Am. J. Hum. Genet..

[15] Liu Y., Lv Y., Zarrei M., Dong R., Yang X., Higginbotham E.J., Li Y., Zhao D., Song F., Yang Y. (2022). Chromosomal Mi-croarray Analysis of 410 Han Chinese Patients with Autism Spectrum Disorder or Unexplained Intellectual Disability and Developmental Delay. NPJ Genom. Med..

[16] Huang J., Liu J., Tian R., Liu K., Zhuang P., Sherman H.T., Budjan C., Fong M., Jeong M.-S., Kong X.-J. (2021). A Next Generation Sequencing-Based Protocol for Screening of Variants of Concern in Autism Spectrum Disorder. Cells.

[17] Hiraide T., Yamoto K., Masunaga Y., Asahina M., Endoh Y., Ohkubo Y., Matsubayashi T., Tsurui S., Yamada H., Yanagi K. (2021). Genetic and Phenotypic Analysis of 101 Patients with Developmental Delay or Intellectual Disability Using Whole-Exome Sequencing. Clin. Genet..

[18] Kreyszig E. (1979). Advanced Engineering Mathematics.

[19] Nguyen T.T., Van Tran K., Ho T.C., Nguyen H.X., Nguyen T.T. (2024). A systematic analysis with the hierarchical cluster analysis strategy on the complex interaction of TERT and CTNNB1 somatic mutations in Vietnamese hepatocellular carcinoma patients. Gene.

[20] Nguyen T.T., Ho T.C., Bui H.T.T., Tran V.-K., Nguyen T.T. (2024). Multi-clustering study on the association between human leukocyte antigen-DP-DQ and hepatitis B virus-related hepatocellular carcinoma and cirrhosis in Vietnam. World J. Gastroenterol..

[21] Nguyen T.T., Ho C.T., Bui H.T.T., Ho L.K., Ta V.T. (2023). Multidimensional Machine Learning for Assessing Parameters Associated With COVID-19 in Vietnam: Validation Study. JMIR Form. Res..

[22] Ngoc Tram H.T., Thu Huong B.T., Duc Hinh N., Thuy P.T., Lan Anh L.T., Kim Phuong D.T., Thu Lan H., Cam Tu H., Koerber N., Bauer T. (2023). Multidimensional Analysis of the Mother-to-child Transmission Risk Factors in Chronic Hepatitis B Virus Infection in Pregnant Women in Vietnam. Clin. Ter..

[23] Bui H.T.T., Phương Q.N.T., Tu H.C., Phuong S.N., Pham T.T., Vu T., Thu H.N.T., Ho L.K., Tien D.N. (2024). The Roles of NOTCH3 p.R544C and Thrombophilia Genes in Vietnamese Patients With Ischemic Stroke: Study Involving a Hierarchical Cluster Analysis. JMIR Bioinform. Biotechnol..

[24] Dung N.T., Quynh N.-T.P., Tu H.C., Huong B.-T.T., Thuy P.-T., Thu V.-T.H., Trong L.M., Lam H.K., Sinh N.P. (2024). Stochastic Petri net model with random time of Vietnamese ischemic stroke patient treatment process. Ital. J. Med..

[25] Dung N., Huong B., Tu H., Tram H. (2024). Direct Reciprocal Interaction Between Platelet Count and HBeAg Status in HBsAg-positive Pregnant Women. Acta Inf. Inform. Med..

[26] Tien D.N., Bui H.T.T., Ngoc T.H.T., Pham T.T., Nguyen D.T., Thu H.N.T., Vu T.T.H., Luong T.L.A., Hoang L.T., Tu H.C. (2025). A Data-Driven Approach to Assessing Hepatitis B Moth-er-to-Child Transmission Risk Prediction Model: Machine Learning Perspective. JMIR Form. Res..

[27] Klouwer F., Meester-Delver A., Vaz F., Waterham H., Hennekam R., Poll-The B. (2017). Development and validation of a severity scoring system for Zellweger spectrum disorders. Clin. Genet..

[28] Aglan M.S., Hosny L., El-Houssini R., Abdelhadi S., Salem F., ElBanna R.A.S., Awad S.A., Zaki M.E., Temtamy S.A. (2012). A scoring system for the assessment of clinical severity in osteogenesis imperfecta. J. Child. Orthop..

[29] Nagy D., Verheyen S., Wigby K.M., Borovikov A., Sharkov A., Slegesky V., Larson A., Fagerberg C., Brasch-Andersen C., Kibæk M. (2022). Genotype-Phenotype Comparison in POGZ-Related Neurodevelopmental Disorders by Using Clinical Scoring. Genes.

[30] A Braun D., Rao J., Mollet G., Schapiro D., Daugeron M.-C., Tan W., Gribouval O., Boyer O., Revy P., Jobst-Schwan T. (2017). Mutations in KEOPS-complex genes cause nephrotic syn-drome with primary microcephaly. Nat. Genet..

[31] Karaca E., Harel T., Pehlivan D., Jhangiani S.N., Gambin T., Coban Akdemir Z., Gonzaga-Jauregui C., Erdin S., Bayram Y., Campbell I.M. (2015). Genes that affect brain structure and function identified by rare variant analyses of Mendelian neurologic disease. Nat. Genet..

[32] Stenson P.D., Mort M., Ball E.V., Evans K., Hayden M., Heywood S., Hussain M., Phillips A.D., Cooper D.N. (2017). The Human Gene Mutation Database: Towards a Comprehensive Repository of Inherited Mutation Data for Medical Research, Genetic Diagnosis and next-Generation Sequencing Studies. Hum. Genet..

[33] Tran T.T., Hoang T.H., Tran M.H., Nguyen N.T., Nguyen D.T., Pham T.M., Nguyen N.N., Vu G.M., Duong V.C., Vu Q.T. (2025). VN1K: A genome graph-based and function-driven multi-omics and phenomics resource for the Vietnamese population. bioRxiv.

[34] Werling D.M., Geschwind D.H. (2013). Sex differences in autism spectrum disorders. Curr. Opin. Neurol..

[35] Curtin S.C., Xu F., Gilbert T., Anderson R.N. (2023). The Prevalence of Intellectual Disability Varied by Sex, Age Group, and Race and Hispanic Origin. National Center for Health Statistics. https://www.cdc.gov/nchs/products/databriefs/db473.htm.

[36] Louw N., Carstens N., Lombard Z. (2023). Incorporating CNV Analysis Improves the Yield of Exome Sequencing for Rare Monogenic Disorders-an Important Consideration for Resource-Constrained Settings. Front. Genet..

[37] McRae J.F., Clayton S., Fitzgerald T.W., Kaplanis J., Prigmore E., Rajan D., Sifrim A., Aitken S., Akawi N., Alvi M. (2017). Prevalence and Architecture of de Novo Mutations in Developmental Disorders. Nature.

[38] Lemke J.R., Riesch E., Scheurenbrand T., Schubach M., Wilhelm C., Steiner I., Hansen J., Courage C., Gallati S., Bürki S. (2012). Targeted next Generation Sequencing as a Diagnostic Tool in Epileptic Disorders. Epilepsia.

[39] van Wegberg A.M.J., MacDonald A., Ahring K., Bélanger-Quintana A., Blau N., Bosch A.M., Burlina A., Campistol J., Feillet F., Giżewska M. (2017). The complete European guidelines on phenylketonuria: Diagnosis and treatment. Orphanet J. Rare Dis..

[40] Richards S., Aziz N., Bale S., Bick D., Das S., Gastier-Foster J., Grody W.W., Hegde M., Lyon E., Spector E. (2015). Standards and Guidelines for the Interpretation of Sequence Variants: A Joint Consensus Recommendation of the American College of Medical Genetics and Genomics and the Association for Molecular Pathology. Genet. Med..

[41] Riggs E.R., Andersen E.F., Cherry A.M., Kantarci S., Kearney H., Patel A., Raca G., Ritter D.I., South S.T., Thorland E.C. (2020). Technical standards for the interpretation and reporting of constitutional copy-number variants: A joint consensus recommendation of the American College of Medical Genetics and Genomics (ACMG) and the Clinical Genome Resource (ClinGen). Genet. Med..

[42] Major  R.M., Juengst E.T. (2025). Prenatal gene editing for neurodevelopmental diseases: Ethical considerations. Am. J. Hum. Genet..

[43] Alamowitch S., Plaisier E., Favrole P., Prost C., Chen Z., Van Agtmael T., Marro B., Ronco P. (2009). Cerebrovascular disease related to COL4A1 mutations in HANAC syndrome. Neurology.

