# Supplementary material for: Maximizing Diagnostic Yield in Intellectual Disability Through Exome Sequencing: Genotype–Phenotype Insights in a Vietnamese Cohort
Source: Diagnostics (Basel). 2025 Nov 7;15(22):2821. doi: 10.3390/diagnostics15222821 (PMC12651281; doi:10.3390/diagnostics15222821)
Supplement: Supplementary file 1 [file diagnostics-15-02821-s001.zip › Supplementary Figures.pdf]

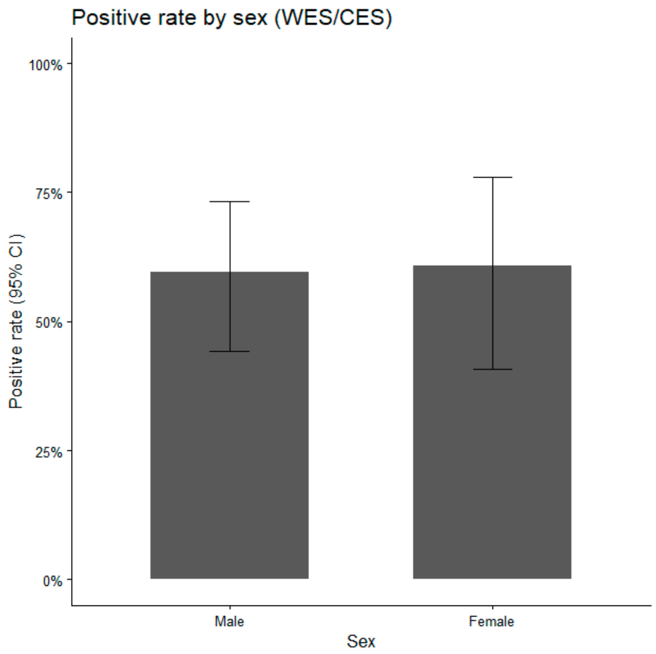

Figure S1. Positive rate by sex among patients tested with WES/CES.
Bar chart illustrating the proportion of patients with positive molecular findings (pathogenic or likely pathogenic variants) stratified by sex. Error bars indicate 95% confidence intervals (CI). No statistically significant difference was observed between males and females ($p > 0.05$, Chi-square or Fisher's exact test).

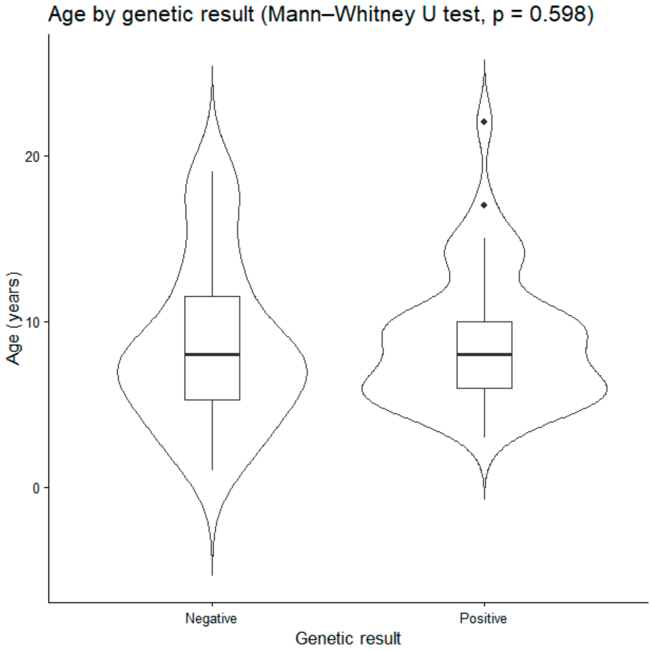

Figure S2. Comparison of age between patients with positive and negative genetic findings.

Violin and box plots showing age distribution by genetic test results; no significant difference observed (ns; Mann–Whitney U test, p = 0.598).

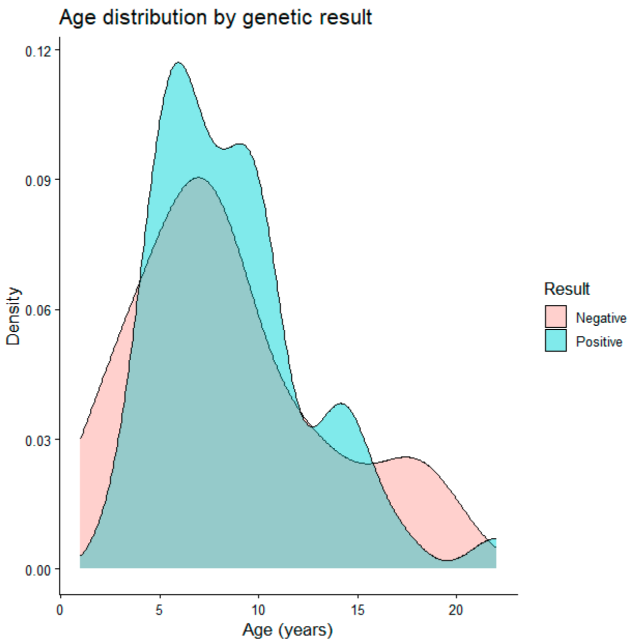

Figure S3. Age distribution by genetic test result.

Density plot illustrating the age distribution of patients according to genetic testing outcomes. Substantial overlap between positive and negative groups indicates no apparent age-related bias in diagnostic yield.

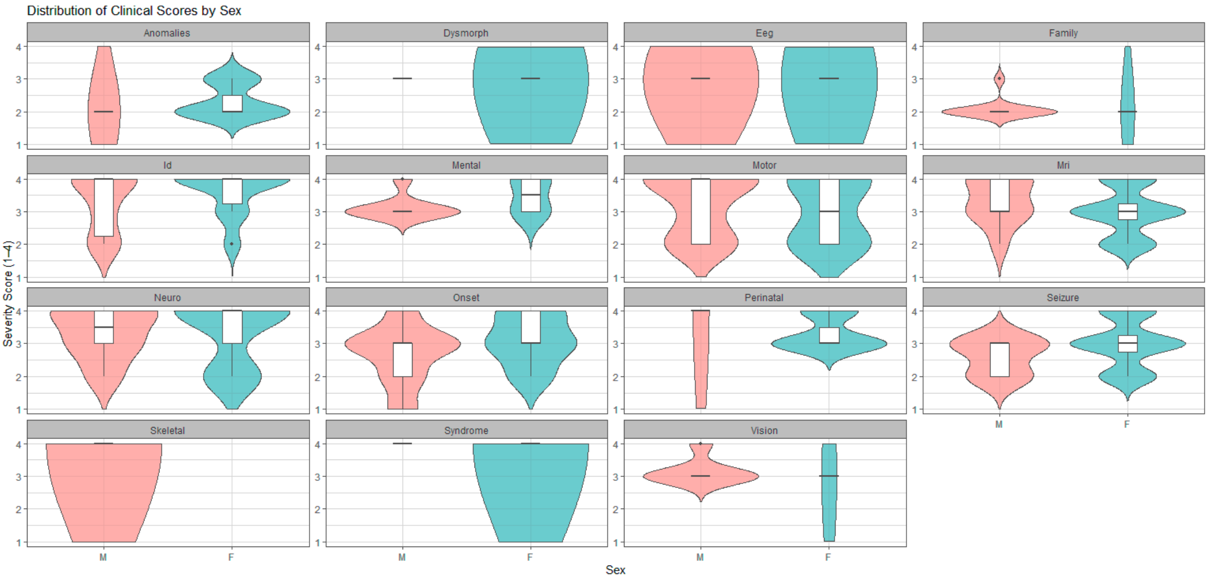

Figure S4: Distribution of clinical scores by sex.

Violin and box plots showing the distribution of semi-quantitative clinical severity scores (1–4) across 15 domains, stratified by sex. Each subplot represents a phenotypic domain (e.g., onset, vision, neurological, intellectual disability, MRI findings). Male (M) and female (F) groups are shown in distinct colors. Boxes indicate interquartile ranges (IQR), with central lines representing median scores.

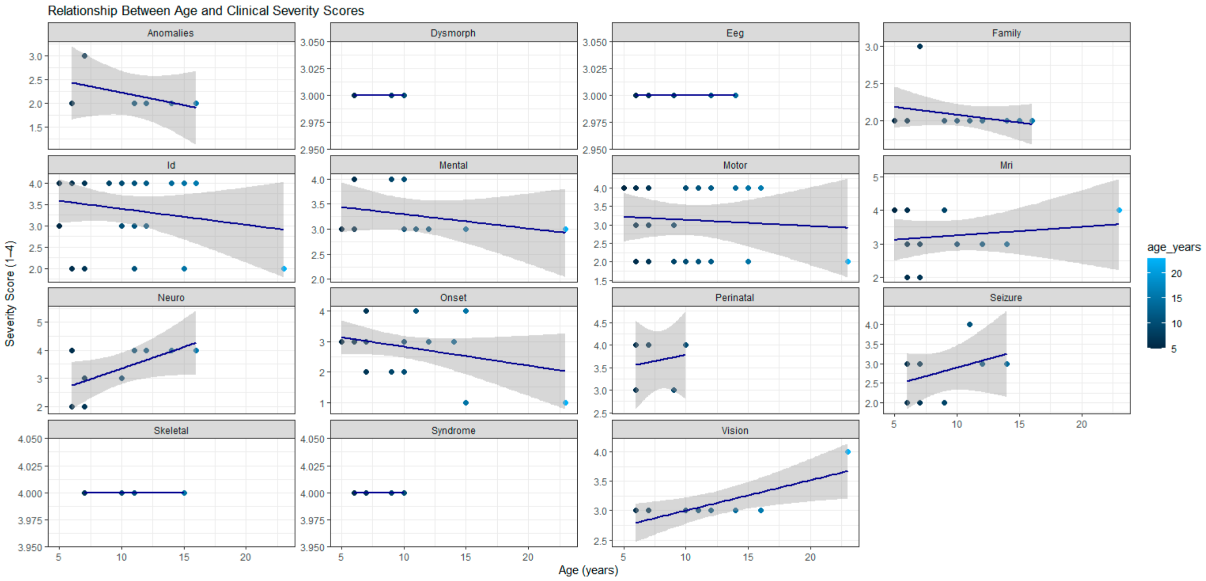

Figure S5: Relationship between age and clinical severity scores.

Scatter plots with fitted linear regression lines showing the relationship between patient age (x-axis) and clinical severity scores (1–4, y-axis) across 15 phenotypic domains. Each point represents an individual patient, color-coded by age in years. Shaded areas indicate 95% confidence intervals of the regression lines.

```
# A tibble: 18 × 4
   gene     `1`   `3`   `2`
   <chr>  <int> <int> <int>
 1 ACTG1      1     0     0
 2 ADSL       0     1     0
 3 ATP1A3     1     0     0
 4 CHD3       1     0     0
 5 GNPAT      2     0     0
 6 HNRNPU     0     1     0
 7 IQSEC2     1     0     0
 8 KCNMA1     0     1     0
 9 NGLY1      1     0     0
10 NIPBL      1     0     0
11 NKX6-2     5     0     0
12 PAH        0     2     0
13 PGAP3      2     0     0
14 PLP1       2     0     0
15 POLR1C     0     1     0
16 SCN2A      1     2     0
17 SMAD6      1     0     0
18 TCF4       0     0     2
```

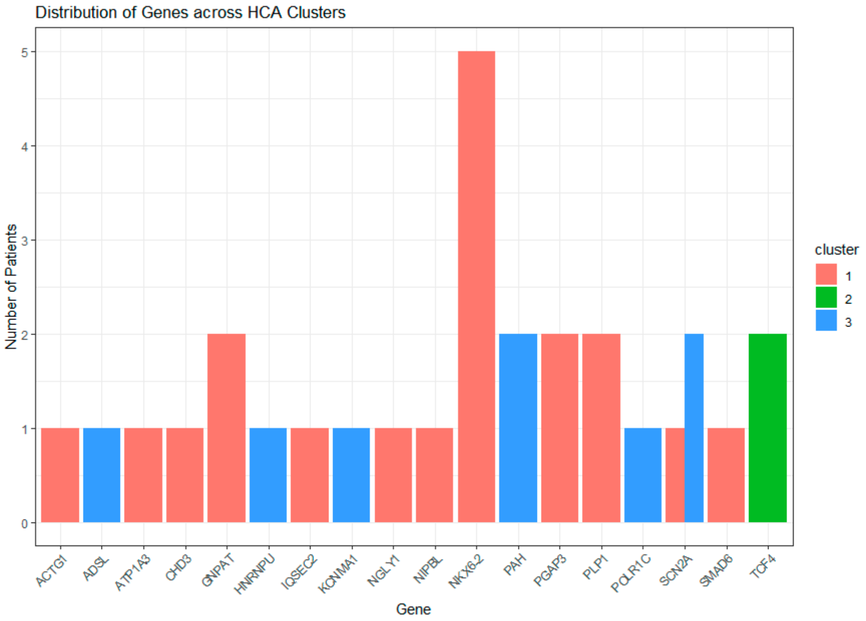

Figure S6. Frequency of genes across HCA-derived clusters.
Cluster 1 (red) contains the largest number of genes (12/18), predominantly transcriptional, metabolic, and myelination-related genes, including NKX6-2, PLP1, PGAP3, and CHD3. Cluster 2 (green) is mainly characterized by epilepsy- and ion channel–related genes, such as SCN2A and PAH. Cluster 3 (blue) contains few genes, representing intermediate or mixed phenotypes, with occasional overlap (e.g., TCF4 and PAH). These frequencies demonstrate that the HCA-derived clusters reflect underlying biological pathway coherence rather than random grouping. For example, NKX6-2 (myelination) was the most frequent gene in Cluster 1 ($n = 5$), corresponding to the severe neurodevelopmental phenotype observed in this group.

```
        Pearson's Chi-squared test

data:  tab
X-squared = 54.566, df = 34, p-value = 0.0141


Expected counts:

                1          2         3
  ACTG1  0.6551724 0.06896552 0.2758621
  ADSL   0.6551724 0.06896552 0.2758621
  ATP1A3 0.6551724 0.06896552 0.2758621
  CHD3   0.6551724 0.06896552 0.2758621
  GNPAT  1.3103448 0.13793103 0.5517241
  HNRNPU 0.6551724 0.06896552 0.2758621
  IQSEC2 0.6551724 0.06896552 0.2758621
  KCNMA1 0.6551724 0.06896552 0.2758621
  NGLY1  0.6551724 0.06896552 0.2758621
  NIPBL  0.6551724 0.06896552 0.2758621
  NKX6-2 3.2758621 0.34482759 1.3793103
  PAH    1.3103448 0.13793103 0.5517241
  PGAP3  1.3103448 0.13793103 0.5517241
  PLP1   1.3103448 0.13793103 0.5517241
  POLR1C 0.6551724 0.06896552 0.2758621
  SCN2A  1.9655172 0.20689655 0.8275862
  SMAD6  0.6551724 0.06896552 0.2758621
  TCF4   1.3103448 0.13793103 0.5517241
Warning message:
In stats::chisq.test(x, y, ...) :
  Chi-squared approximation may be incorrect
```

Figure S7. Pearson's Chi-squared test revealed a statistically significant association between gene distribution and hierarchical clusters (χ^2 = 54.566, df = 34, p = 0.0141), indicating that gene occurrences were not randomly distributed across clusters. This finding supports the biological validity of the clustering, with specific genes (e.g., NKX6-2, PLP1, PGAP3) concentrated within transcriptional/myelination-related clusters, while others (e.g., SCN2A, PAH) grouped within ion-channel or metabolic pathways.

```
# A tibble: 15 × 4
   pathway                        `3`   `1`   `2`
   <chr>                        <int> <int> <int>
 1 Amino acid metabolism            2     0     0
 2 Chromatin remodeling             0     1     0
 3 Cohesin complex                  0     1     0
 4 Cytoskeleton / Cell structure    0     1     0
 5 GPI-anchor metabolism            0     2     0
 6 Ion channel                      3     2     0
 7 Metabolic pathway                1     0     0
 8 Myelination / Oligodendrocyte    0     7     0
 9 Peroxisomal metabolism           0     2     0
10 Protein degradation              0     1     0
11 RNA processing                   1     0     0
12 Synaptic signaling               0     1     0
13 TGF-beta signaling               0     1     0
14 Transcription / Translation      1     0     0
15 Transcription factor             0     0     2

        Pearson's Chi-squared test

data:  tab_path
X-squared = 51.818, df = 28, p-value = 0.004025

Warning message:
In stats::chisq.test(x, y, ...) :
  Chi-squared approximation may be incorrect
```

Figure S8. Pathway-level distribution across HCA-derived clusters.
The table summarizes the distribution of functional pathways among the three hierarchical cluster analysis (HCA)–derived clusters. Each value represents the number of genes assigned to a specific biological pathway within a given cluster. Distinct enrichment patterns were observed: Cluster 1 was dominated by myelination and transcriptional pathways, Cluster 2 by transcription factor–related genes, and Cluster 3 by ion channel and metabolic processes. This distribution supports the biological coherence of the clusters and reflects functional heterogeneity of the underlying monogenic variants. The pathway distributions differed significantly across clusters, confirming that HCA captured biologically distinct mechanisms rather than random overlap.
