# Supplementary material for: Maximizing Diagnostic Yield in Intellectual Disability Through Exome Sequencing: Genotype–Phenotype Insights in a Vietnamese Cohort
Source: Diagnostics (Basel). 2025 Nov 7;15(22):2821. doi: 10.3390/diagnostics15222821 (PMC12651281; doi:10.3390/diagnostics15222821)
Supplement: Supplementary file 1 [file diagnostics-15-02821-s001.zip › Supplementary Table S1.pdf]

Supplemetary Table S1: Monogenic variants

	ID	Gene	Age	Sex	RefSeq	DNA Change	Amino Acid Change	Type	Zyg	Class	PMID	dbSNP ID	gnomAD MAF	MOI	Parental Origin	TEST
**Confirmed cases**																
**1**	ID1.1	NKX6-2	2014	F	NM_177400.3	c.234dup	p.Leu79CysfsTer109	Frameshift	HOM	P	-	rs765650727	0.00003	AR	MAT, PAT	WES
**2**	ID1.2	NKX6-2	2009	M	NM_177400.3	c.234dup	p.Leu79CysfsTer109	Frameshift	HOM	P	-	rs765650728	0.00003	AR	MAT, PAT	Sanger
**3**	ID2.1	NKX6-2	2019	M	NM_177400.3	c.234dup	p.Leu79CysfsTer109	Frameshift	HOM	P	-	rs765650729	0.00003	AR	MAT, PAT	WES
**4**	ID2.2	NKX6-2	2013	M	NM_177400.4	c.234dup	p.Leu79CysfsTer109	Frameshift	HOM	P	-	rs765650730	0.00003	AR	MAT, PAT	Sanger
**5**	ID2.3	NKX6-2	2011	F	NM_177400.5	c.234dup	p.Leu79CysfsTer109	Frameshift	HOM	P	-	rs765650731	0.00003	AR	MAT, PAT	Sanger
**6**	ID3	TCF4	2019	F	NM_001083962.2	c.912_913insTACT	p.Ser305TyrfsTer2	Frameshift	HET	P	-	-	-	AD	De novo	WES
**7**	ID4	TCF4	2016	F	NM_001083962.2	c.514_517del	p.Lys172PhefsTer61	Frameshift	HET	P	25167861,18728071,22045651	rs398123561	-	AD	De novo	WES
**8**	ID5	SCN2A	2014	F	NM_001040142.2	c.2659G>C	p.Val887Leu	Missense	HET	LP	-	rs1574641605	-	AD	N/A	CES
**9**	ID6.1	SCN2A	2010	M	NM_001040142.2	c.914_923dup	p.Arg309TyrfsTer4	Frameshift	HET	LP	-	-	-	AD	De novo	CES

**10**	ID6.2	SCN2A	2014	M	NM_001040142.2	c.914_923dup	p.Arg309TyrfsTer4	Frameshift	HET	LP	-	-	-	AD	De novo	CES
**11**	ID7	PAH	2013	F	NM_000277.3	c.1200-1G>C	(p.?)	Splice acceptor	HOM	P	31102715, 30747360, 28915855, 26503515	rs62507322	-	AR	PAT, MAT	CES
**12**	ID8	PAH	2015	M	NM_000277.3	c.722del	p.Arg241ProfsTer100	Frameshift	HOM	P	30459323, 26322415,16256386, 25456745,12905706, 1301187, 9634518, 25550961, 29499199	rs199475657	-	AR	PAT, MAT	WES
**13**	ID9	IQSEC2	2018	M	NM_001111125.3	c.4419dup	p.Ser1474ValfsTer21	Frameshift	HEM	P	30666632, 25741868	-	-	XLR	De novo	WES
**14**	ID10	KCNMA1	2018	F	NM_001161352.2	c.3158A>G	p.Asn1053Ser	Missense	HET	P	26195193	rs886039469	-	AD	De novo	WES
**15**	ID11	POLR1C	2002	M	NM_203290.4	c.70-1G>T	(p.?)	Splice acceptor	HET	LP	-	-	-	AR	MAT	WES

	ID11				NM_203290.4	c.943G>T	p.Val315Leu	Missense	HET	LP	-	rs762136709	-		PAT	
**16**	ID12.1	GNPAT	2010	M	NM_014236.4	c.632G>A	p.Arg211His	Missense	HOM	P	9536089, 7530787, 11152660	rs121434439	0.00001	AR	PAT, MAT	WES
**17**	ID12.2	GNPAT	2014	M	NM_014236.5	c.632G>A	p.Arg211His	Missense	HOM	P	9536089, 7530787, 11152660	rs121434440	0.00001	AR	PAT, MAT	Sanger
**18**	ID13.1	PGAP3	2020	M	NM_033419.5	c.827C>T	p.Pro276Leu	Missense	HOM	P	29310717, 35887114, 34582790	rs750093817	0.00006	AR	PAT, MAT	WES
**19**	ID13.2	PGAP3	2014	F	NM_033419.5	c.827C>T	p.Pro276Leu	Missense	HOM	P	29310717, 35887114, 34582790	rs750093818	0.00006	AR	PAT, MAT	Sanger
**20**	ID14	HNRNPU	2019	M	NM_031844.3	c.727_728del	p.Lys243GlufsTer4	Frameshift	HET	LP	-	-	-	AD	De novo	WES
**21**	ID15.1	PLP1	2015	M	NM_000533.3	c.649G>A	p.Gly217Ser	Missense	HEM	P	7679906, 7539212	rs2147766965	-	XLR	MAT	WES
**22**	ID15.2	PLP1	2018	M	NM_000533.3	c.649G>A	p.Gly217Ser	Missense	HEM	P	7679906, 7539212	rs2147766965	-	XLR	MAT	Sanger
**23**	ID16	ADSL	2016	M	NM_000026.4	c.569G>A	p.Arg190Gln	Missense	HET	P	10090474, 10888601, 201279	rs28941471	0.00006	AR	MAT	WES

											76, 22180458					
	ID16				NM_000026.4	c.3G>A	p.Met1Ile	Start loss	HET	LP	-	-	-		PAT	
**24**	ID17	ACTG1	2015	M	NM_001614.5	c.469G>A	p.Asp157Asn	Missense	HET	LP	-	-	-	AD	De novo	WES
**25**	ID18	ATP1A3	2019	F	NM_152296.5	c.2443G>A	p.Glu815Lys	Missense	HET	P	25996915, 22842232, 26410222, 28293679, 23409136, 28637637, 22850527, 21911500, 24631656, 30071271, 20301294, 26410222	rs387907281	-	AD	De novo	WES
	ID18	CHD3	2019		NM_001005273.3	c.1744del	p.Gln582SerfsTer28	Frameshift	HET	LP	-	-	-	AD	De novo	WES

**26**	ID19	NIPBL	2018	F	NM_133433.4	c.2602C>T	p.Arg868Ter	Nonsense	HET	P	-	rs398124466	-	AD	N/A	CES
**27**	ID20	NGLY1	2020	M	NM_001145293.2	c.1177C>T	p.Arg393Ter	Nonsense	HOM	LP	24651605, 31965062	rs146140738	0.00040 (A)	AR	MAT, PAT	WES
**28**	ID21	SMAD6	2018	M	NM_005585.5	c.269dup	p.Arg91GlufsTer30	Frameshift	HET	P	28808027	rs1006397889	-	AD	De novo	WES
**Unconfirmed cases**																
**29**	ID22	OSGEP	2017	F	NM_017807.4	c.280C>T	p.Arg94Cys	Missense	HET	VUS	-	rs768795595	0.00001	AR	PAT	WES
	ID22				NM_017807.4	c.143A>G	p.His48Arg	Missense	HET	VUS	-	-	-		MAT	
**30**	ID23	HEPHL1	2009	M	NM_001098672.2	c.2857C>T	p.Arg953Ter	Nonsense	HET	P	-	rs542337286	0.00020 (T)	AR	MAT	WES
	ID23				NM_001098672.2	c.84G>A	p.Thr28=	Synonymous	HET	VUS	-	-	-		PAT	
**31**	ID24	PRUNE1	2014	F	NM_021222.3	c.1066C>T	p.Gln356Ter	Nonsense	HOM	VUS	-	-	-	AR	MAT, PAT	WES
**32**	ID25	COL4A1	2010	F	NM_001845.6	c.4187G>A	p.Gly1396Asp	Missense	HET	LP	-	-	-	AD	PAT	WES
**33**	ID26	ATAD3A	2016	M	NM_001170535.3	c.1759T>C	Ter587Argext*62	Stop loss	HET	LP	-	-	-	AD,AR	MAT	WES
**34**	ID27	MTSS2	2019	M	NM_138383.3	c.1790C>T	p.Thr597Met	Missense	HET	VUS	25558065	rs531163149	0.00060 (A)	AD	PAT	WES
**35**	ID28	MTSS2	2014	F	NM_138383.3	c.1790C>T	p.Thr597Met	Missense	HET	VUS	25558065	rs531163150	0.00060 (A)	AD	PAT	CES
