# Supplementary material for: Maximizing Diagnostic Yield in Intellectual Disability Through Exome Sequencing: Genotype–Phenotype Insights in a Vietnamese Cohort
Source: Diagnostics (Basel). 2025 Nov 7;15(22):2821. doi: 10.3390/diagnostics15222821 (PMC12651281; doi:10.3390/diagnostics15222821)
Supplement: Supplementary file 1 [file diagnostics-15-02821-s001.zip › Supplementary Table S2.pdf]

# Supplementary Table S2: Monogenic variants phenotypes

| ID | Age | Sex | Clinical Diagnosis | Gene | Onset characteristics | Perinatal history | Vision/Eyes | Neurological/ neuromuscular | Seizures (Onset Age) | EEG | Brain MRI | Mental disorder | Motor development | Intelectual Disability | Dysmorphism | Skeletal | Accompanying Anomalies or Conditions | Family history | Syndrome/ Non-syndrome |
|---|---|---|---|---|---|---|---|---|---|---|---|---|---|---|---|---|---|---|---|
| ID1.1 | 2014 | F | Cerebral palsy | NKX6-2 | Nystagmus (1 Mo) | Normal | Abnormal eyes movement | Dystonia, spastic tetraplegia | No | Not done | Not done | No | Loss of ambulation | Severe/profound delay | No | Normal | frequent respiratory illnesses | Yes | No |
| ID1.2 | 2009 | M | Cerebral palsy | NKX6-2 | Nystagmus (3 Mo) | Normal | Abnormal eyes movement | Dystonia, spastic tetraplegia | No | Not done | Not done | No | Loss of ambulation | Severe/profound delay | No | Normal | frequent respiratory illnesses | Yes | No |
| ID2.1 | 2019 | M | Cerebral palsy | NKX6-2 | Nystagmus, Head lag (4 Mo) | Normal | Abnormal eyes movement | Dystonia, spastic tetraplegia | Tonic seizures (1yo) | Abnormal | white matter degeneration | No | Loss of ambulation | Severe/profound delay | No | Normal | frequent respiratory illnesses | Yes | No |
| ID2.2 | 2013 | M | Cerebral palsy | NKX6-2 | Nystagmus, Head lag (4 Mo) | Normal | Abnormal eyes movement | Dystonia, spastic tetraplegia | Tonic seizures (1yo) | Abnormal | white matter degeneration | No | Loss of ambulation | Severe/profound delay | No | Normal | frequent respiratory illnesses | Yes | No |
| ID2.3 | 2011 | F | Cerebral palsy | NKX6-2 | Nystagmus, Head lag (4 Mo) | Normal | Abnormal eyes movement | Dystonia, spastic tetraplegia | Tonic seizures (1yo) | Abnormal | white matter degeneration | No | Loss of ambulation | Severe/profound delay | No | Normal | frequent respiratory illnesses | Yes | No |
| ID3 | 2019 | F | Developmental delay | TCF4 | Unable to roll over and sit independently (8 Mo) | Lateral ventriculomegaly | No | No | No | No | Ventriculomegaly, depicting various sulci (9 Mo) | aggressive behavior | Severe delay (Crawling) | Severe delay (Unable to speak) | Protruding lower face, full cheeks, thick, fleshy lips, broad nasal bridge, widely spaced teeth | Normal | | No | Pitt-Hopkins syndrome |
| ID4 | 2016 | F | Developmental delay | TCF4 | 12 Mo | Oligohydramnios at 39 weeks | No | No | No | Not done | normal (22 Mo) | aggressive behavior | Moderate delay (Walk independently at 33 Mo) | Severe delay | Coarse face, Protruding lower face, full cheeks, thick, fleshy lips, broad nasal bridge, widely spaced teeth | Normal | | No | Pitt-Hopkins syndrome |
| ID5 | 2014 | F | Epilepsy and developmental delay | SCN2A | Seizure (1 Mo) | Normal | No | No | 1 Mo | N/A | Normal | No | Loss of ambulation | Severe delay | No | Normal | | No | No |

| | | | | | | | | | | | | | | | | | | | |
|---|---|---|---|---|---|---|---|---|---|---|---|---|---|---|---|---|---|---|---|
| ID6.1 | 2010 | M | ID, ASD | SCN2A | Speech selay (3 Yo) | Normal | No | No | No | Normal | N/A | ASD | Mild delay (poor motor coordination) | Mild delay | No | Normal | | Yes | No |
| ID6.2 | 2014 | M | ID, ASD | SCN2A | 3yrs (Speech selay) | Normal | No | No | No | Normal | N/A | ASD | Mild delay (poor motor coordination) | Mild delay | No | Normal | | Yes | No |
| ID7 | 2013 | F | ID, ASD | PAH | Head lag (7 Mo) | Normal | No | No | No | Normal | Delay myelination | ASD | Mild delay | Moderate delay | Yes | Normal | HPA | No | No |
| ID8 | 2015 | M | ID, ASD | PAH | walking delay (2 Yo) | Normal | No | No | No | Not done | Not done | ASD | Mild delay | Moderate delay | Yes | Normal | HPA | - | No |
| ID9 | 2018 | M | Developmental delay, epilepsy | IQSEC2 | Head lag (4 Mo) | Normal | No | Seizure | 1 yo | Abnormal | Lateral ventriculomegaly | No | Severe delay | Severe delay | No | Normal | | No | No |
| ID10 | 2018 | F | Epilepsy and developmental delay | KCNMA1 | speech delay (20 Mo) | Normal | No | Seizure | 13 Mo | Abnormal | Not done | N/A | Mild delay | Mild delay | No | Normal | | No | No |
| ID11 | 2002 | M | Bệnh não chất trắng | POLR1C | hand tremor (5 Yo) | Normal | Myopia (Vision: 1/10) | No | No | Not done | Vanishing white matter, Cerebellar atrophy | ASD | Mild delay | Mild delay | No | Normal | | - | No |
| ID12.1 | 2010 | M | Developmental delay, cataracts, chondrodysplasia | GNPAT | newborn | Normal | Yes | Hypetonia | No | Not done | Not done | No | Loss of ambulation | Severe delay | Yes | Contractures, rotational malalignment | | Yes | No |
| ID12.2 | 2014 | M | Developmental delay, cataracts, chondrodysplasia | GNPAT | newborn | Normal | Yes | Hypetonia | No | Not done | Not done | No | Loss of ambulation | Severe delay | Yes | Contractures, rotational malalignment | | Yes | No |
| ID13.1 | 2020 | M | Intelectual disability, autism | PGAP3 | Speech delay | Normal | No | No | N/A | ? | Not done | ASD, ADHD | Delay fine motor development | Moderate delay | No | Normal | Clef palate | Yes | No |
| ID13.2 | 2014 | F | Intelectual disability, autism | PGAP3 | Speech delay | Normal | No | No | N/A | ? | Not done | ASD, ADHD | Delay fine motor development | Moderate delay | No | Normal | No | Yes | No |
| ID14 | 2019 | M | Developmental delay, epilepsy | HNRNPU | 5Mo (Febrile seizure) | Normal | No | No | Febrile seizure ( 5Mo) | Abnormal | nonspecific subcortical white matter lesions in the bilateral parietal lobes. | ASD, ADHD | Mild delay | Mild delay | No | Normal | No | Yes | No |

| | | | | | | | | | | | | | | | | | | | |
|---|---|---|---|---|---|---|---|---|---|---|---|---|---|---|---|---|---|---|---|
| ID15.1 | 2015 | M | Cerebral palsy/Spastic paraplegia | PLP1 | Head lag (4 Mo) | Abnormal Ultrasound | strabismous | Spastic paraplegia | No | Not done | Delay myelination | No | Loss of ambulation | Severe delay | No | Muscle atrophy, joint contractures,talipes equinovarus) | | Yes | No |
| ID15.2 | 2018 | M | Cerebral palsy/Spastic paraplegia | PLP1 | Head lag (4 Mo) | N/A | strabismous | Spastic paraplegia | No | Not done | Delay myelination | No | Loss of ambulation | Severe delay | No | Muscle atrophy, joint contractures,talipes equinovarus) | | Yes (Older sister) | No |
| ID16 | 2016 | M | Developmental delay | ADSL | Walking difficulties ( 2 Yo) | Normal | Normal | No | Yes (2 yo) | abnormal | vanishing white matter | N/A | Severe delay | Severe delay | No | Normal | | Yes | No |
| ID17 | 2015 | M | ID, Dandy Walker syndrome | ACTG1 | Delay rolling over (5Mo) | Abnormal ultrasound : Dandy Walker syndrome | Normal | Normal | No | Not done | Dandy walker syndrome | ADHD, aggressive behaviour | fine motor disorder | moderate delay | Yes (Long palpebral fissures, hypertelorism) | Muscle weekness | | No | Baraitser - Winter |
| ID18 | 2019 | F | Bại não | ATP1A3 | Delay rolling over (5Mo) | mother had appendectomy at 4 months pregnant, abnormal ultrasound : hydrocephalus | Normal | Hypertonia,episodic hemiplegia | Nocturnal Seizures (1 Yo) | N/A | Hydrocephalus | N/A | Loss of ambulation | Severe delay | Yes ( Prominent forehea, Frontal bossing, Full cheeks, Pointed chin) | Normal | | No | No |
| | | | | CHD3 | | | | | | | | | | | | | | No | Snijders Blok-Campeau syndrome |
| ID19 | 2018 | F | ID, ASD | NIPBL | Newborn, dysmorphy | Normal | Normal | N/A | No | Not done | Not done | NA | Moderate delay | Severe delay | Yes | Normal | Vesicoureteral reflux | No | Cornelia De lange |
| ID20 | 2020 | M | Intellectual disability | NGLY1 | Head lag (4 Mo) | Normal | No | Hypotonia | No | Normal | Ventriculomegaly, bilateral temporal subarachnoid space enlargement, delay myelination, | NA | Loss of ambulation | Severe delay | Yes | | Ectopic testicles | No | No |
| ID21 | 2018 | M | Cerebral palsy | SMAD6 | Delay speech, delay walkiing (19 Mo) | abnormal ultrasound (lateral ventriculomegaly, enlarged cisterna magna) | No | Hypertonia | No | Not done | Not done | | Gross and fine motor delays | Mild delay (Speech delay) | Yes | Premature craniosynostosis, hip contracture | | No - | No |
