# Supplementary material for: Maximizing Diagnostic Yield in Intellectual Disability Through Exome Sequencing: Genotype–Phenotype Insights in a Vietnamese Cohort
Source: Diagnostics (Basel). 2025 Nov 7;15(22):2821. doi: 10.3390/diagnostics15222821 (PMC12651281; doi:10.3390/diagnostics15222821)
Supplement: Supplementary file 1 [file diagnostics-15-02821-s001.zip › Supplementary Table S3.pdf]

Supplementary Table S3: Sum up the result of 75 cases

Number	Birth	Gender	Test	Result
1	2006	M	WES	Negative
2	2012	M	WES	Negative
3	2015	M	WES	Negative
4	2017	F	WES	Negative
5	2011	M	WES	Negative
6	2014	F	WES	Negative
7	2016	M	WES	Negative
8	2005	M	WES	Negative
9	2012	M	WES	Negative
10	2006	M	WES	Negative
11	2016	F	WES	Negative
12	2017	M	WES	Negative
13	2018	M	WES	Negative
14	2022	M	WES	Negative
15	2018	F	WES	Negative
16	2007	F	WES	Negative
17	2016	F	WES	Negative
18	2017	F	WES	Negative
19	2018	M	WES	Negative
20	2019	M	WES	Negative
21	2021	F	WES	Negative
22			WES	Negative
23	2016	M	WES	Negative
24	2010	M	WES	Negative<br>Negative
25	2020	M	WES	Negative
26	2016	M	WES	Negative
27	2021	F	WES	Negative
28	2019	M	WES	Negative
29	2021	F	WES	Negative
30	2016	M	WES	Negative
31	2014	F	WES	Positive
32	2009	M	Sanger	Positive
33	2019	M	WES	Positive
34	2013	M	Sanger	Positive
35	2011	F	Sanger	Positive
36	2019	F	WES	Positive
37	2016	F	WES	Positive
38	2014	F	CES	Positive
39	2010	M	CES	Positive
40	2014	M	CES	Positive
41	2013	F	CES	Positive
42	2015	M	WES	Positive
43	2018	M	WES	Positive
44	2018	F	WES	Positive
45	2002	M	WES	Positive
46	2010	M	WES	Positive
47	2014	M	Sanger	Positive
48	2020	M	WES	Positive
49	2014	F	Sanger	Positive
50	2019	M	WES	Positive
51	2015	M	WES	Positive

Supplementary Table S3: Sum up the result of 75 cases

Number	Birth	Gender	Test	Result
52	2018	M	Sanger	Positive
53	2016	M	WES	Positive
54	2015	M	WES	Positive
55	2019	F	WES	Positive
56	2018	F	CES	Positive
57	2020	M	WES	Positive
58	2018	M	WES	Positive
59	2017	F	WES	Positive
60	2009	M	WES	Positive
61	2014	F	WES	Positive
62	2010	F	WES	Positive
63	2016	M	WES	Positive
64	2019	M	WES	Positive
65	2014	F	CES	Positive
66	2019	F	CES	Positive
67	2018	M	WES	Positive
68	2018	M	WES	Positive
69	2007	F	WES	Positive
70	2017	M	CES	Positive
71	2018	M	CES	Positive
72	2016	M	WES	Positive
73	2017	M	CES	Positive
74	2015	F	WES	Positive
75	2021	M	WES	Positive
